# Does Positive Selection Drive Transcription Factor Binding Site Turnover? A Test with Drosophila Cis-Regulatory Modules

**DOI:** 10.1371/journal.pgen.1002053

**Published:** 2011-04-28

**Authors:** Bin Z. He, Alisha K. Holloway, Sebastian J. Maerkl, Martin Kreitman

**Affiliations:** 1 Department of Ecology and Evolution, The University of Chicago, Chicago, Illinois, United States of America; 2 Gladstone Institute, University of California San Francisco, San Francisco, California, United States of America; 3 Laboratory of Biological Network Characterization (LBNC), École Polytechnique Fédérale de Lausanne, Lausanne, Switzerland; 4 Committee on Genetics, Genomics, and Systems Biology, The University of Chicago, Chicago, Illinois, United States of America; Stanford University, United States of America

## Abstract

Transcription factor binding site(s) (TFBS) gain and loss (i.e., turnover) is a well-documented feature of cis-regulatory module (CRM) evolution, yet little attention has been paid to the evolutionary force(s) driving this turnover process. The predominant view, motivated by its widespread occurrence, emphasizes the importance of compensatory mutation and genetic drift. Positive selection, in contrast, although it has been invoked in specific instances of adaptive gene expression evolution, has not been considered as a general alternative to neutral compensatory evolution. In this study we evaluate the two hypotheses by analyzing patterns of single nucleotide polymorphism in the TFBS of well-characterized CRM in two closely related Drosophila species, *Drosophila melanogaster* and *Drosophila simulans*. An important feature of the analysis is classification of TFBS mutations according to the direction of their predicted effect on binding affinity, which allows gains and losses to be evaluated independently along the two phylogenetic lineages. The observed patterns of polymorphism and divergence are not compatible with neutral evolution for either class of mutations. Instead, multiple lines of evidence are consistent with contributions of positive selection to TFBS gain and loss as well as purifying selection in its maintenance. In discussion, we propose a model to reconcile the finding of selection driving TFBS turnover with constrained CRM function over long evolutionary time.

## Author Summary

Transcription factor binding sites (TFBS) turnover (i.e. lineage-specific gain and loss) is a well-documented phenomenon in eukaryote cis-regulatory modules (CRM). The wide spread of the phenomenon and the appearance of conserved expression patterns for diverged orthologous CRM led to the standing view that the observed gain and loss of TFBS were functionally and selectively neutral. To the contrary, genome-wide population genetics analyses have unequivocally identified signatures of positive selection acting in noncoding regions in general, and particularly in 5′ and 3′ untranscribed regions of genes. To specifically test the neutral versus selection hypotheses for the TFBS turnover process, we analyzed natural variation patterns within and between two closely related *Drosophila* species. We found the patterns of divergence and polymorphism for two types of mutations—those inferred to increase or decrease the binding affinity respectively—are not compatible with a neutral hypothesis. Instead, multiple lines of evidence suggested that positive selection has contributed to gain as well as loss of TFBS in the two lineages, with purifying selection maintaining existing TFBS in the population. Spacer sequences also showed signatures of negative and positive selection. We proposed a model of CRM evolution to reconcile the finding of frequent adaptive changes with constraints on long-term evolution.

## Introduction

Gene expression in eukaryotes is generally controlled by transcriptional enhancers, also called cis-regulatory modules (CRM), which are short regions in the genome consisting of a cluster of transcription factor binding sites (TFBS) spaced by intervening sequences (spacers). Individual TFBS have been shown repeatedly to be required for CRM function, yet surprisingly they evolve rapidly and are frequently gained and lost in evolution, attributes that have been demonstrated for a large number of CRM and transcription factors [1]–[5]. These observations pose a challenge to understanding the forces driving the process, especially in cases where CRM function has been preserved despite sequence and structural divergence [6]–[8].

The gain or loss of a TFBS is unlikely to be functionally irrelevant, as repeatedly shown in TFBS knockout experiments [9]–[11], and also demonstrated for the evolved differences between two species by a chimeric enhancer study [12]. One possibility for reconciling conservation of CRM function with rapid TFBS turnover is to assume that each loss of a TFBS is precisely balanced by the simultaneous gain of a cognate TFBS elsewhere in the CRM, a process we will call compensatory evolution [13]. The idea draws on a model first proposed by Kimura [14], where he considers a pair of tightly linked mutant genes that are individually deleterious but in combination restore wildtype function. As applied to TFBS, the gain of a novel site on an allele carrying a mutation that decreases the quality of an existing binding site can offset the mutants fitness cost, creating a selectively neutral double-mutant allele. Binding site turnover - fixation of the double mutant allele - is achieved entirely by genetic drift, thus preserving both CRM function and population fitness. Recently, a theoretical model of this compensatory turnover process was developed to ask about the feasibility of compensatory evolution for TFBS [15]. With plausible assumptions about the mutation rate, population size and selection coefficient on the individual mutations, a completely neutral model cannot achieve a high enough level of turnover to explain *Drosophila* CRM evolution (as exemplified by *eve* stripe 2 enhancer), whereas a model that assumes the double mutant to be more fit than the wildtype does.

This theoretical finding raises the prospects for positive selection being an important driving force of TFBS gain and loss. Instances of directional selection have been documented in cases where a novel regulatory regime is favored [16]. Functional evolution of a transcription factor (TF) can also drive adaptive co-evolution of its TFBS [17]–[19]. Broad-scale studies in noncoding regions and promoters of genes have identified signatures of both selective constraint and positive selection in fruitfly and human [20]–[24]. However, only a small number of population genetics studies have been carried out to specifically test this hypothesis with TFBS or CRM, and because they focus on a single TF or CRM, they have low statistical power to distinguish between neutrality and selection [13]. The generality of the conclusions reached in these studies is also not established [25], [26].

Several different approaches have been designed to detect and quantify selection in the system. One of them has been to consider the genome-wide ensemble of TFBS as evolving at mutation-selection balance, with the fitness of each instance of TFBS being strictly determined by its binding energy [4], [27], [28]. This approach proves useful in studying the strength of selective constraints on functional TFBS. However, the assumption of a unidirectional fitness function, i.e. selection always favors affinity-increasing mutations and against affinity-decreasing ones, could be violated if the loss of a TFBS were favored or gain (or strengthening) of a TFBS is deleterious. Another approach calculates the sum of mutational effects in TFBS on binding affinity and compares it to the expectation under a no-selection model [29]. A higher than expected sum could imply selective removal of affinity-decreasing mutations and therefore the action of purifying selection. Applying this approach to two of the CRM also included in this study, the author provided evidence for purifying selection acting to preserve the functional TFBS in the anterior *Bicoid*-dependent *hunchback* enhancer and the *even-skipped* stripe 2 enhancer. This test can also be used to detect positive selection, although its power is limited due to the mixed signal with purifying selection, which is expected to be dominant in most cases.

In this study, patterns of polymorphism and divergence are investigated in a pair of closely related *Drosophila* species, *D. melanogaster* (*mel*) and *D. simulans* (*sim*). The short evolutionary distance between the two species ensures unambiguous alignment for noncoding sequences and also allows one to capture the potentially rapid dynamics of TFBS gain and loss. A notable challenge in studying TFBS turnover is assembling a high quality set of TFBS that are precisely defined and contain few false positives. Large numbers of potential TFBS can be identified by methods involving genome-wide scans, such as computational prediction or ChIP, but these approaches generally include a large fraction of false positives, thus reducing their attractiveness for investigating the mechanisms of binding site turnover (see Discussion). Instead, we chose to investigate a curated set of high-confidence TFBS identified by DNaseI footprint in well-studied *D. melanogaster* CRM. Short footprint regions usually contain only a single TFBS motif, which, in most cases, could be perfectly aligned with the other species to allow identification of single nucleotide differences within and between the species. Each of these differences, in turn, was evaluated for the predicted magnitude and direction of effect on TF binding energy. The neutral and selection models generate distinguishable predictions in both divergence to polymorphism ratios and in the site frequency spectra. Analysis of these patterns reveal evidence for purifying selection against affinity-decreasing mutations segregating in the population, while multiple lines of evidence indicate positive selection for both gains and losses of TFBS. These empirical findings challenge the prevailing view of neutral compensatory turnover, and have important implications for understanding CRM functional evolution. In the course of the analysis, we also identified and modeled a potential ascertainment that can impact population genetics studies of genomic features that have been identified only in a reference sequence such as TFBS.

## Results

Our analysis focuses on single nucleotide polymorphism (SNP) and divergence in 645 experimentally identified TFBS for 30 transcription factors in 118 autosomal CRM (Table S1), all annotated in REDfly [30]. These 645 TFBS represent the complete set for which we could obtain unambiguous alignment of both within- and between-species sequences without insertion or deletion. We used position weight matrices (PWM) both to identify TFBS within footprints and to predict the magnitude of binding energy differences among variant alleles. Our bioinformatic and experimental validations showed that the PWM used in this study provide reliable and unbiased estimates for the direction of binding affinity change in both *mel* and *sim* (Materials and Methods).

Single nucleotide changes within or between *mel* and *sim* were polarized with outgroup sequences from *D. sechellia*, *D. yakuba* and *D. erecta* using PAML (Materials and Methods). Each derived mutation, therefore, could be categorized with respect to species lineage and to direction of binding affinity change.

### Lineage-specific gain and loss of TFBS as a general pattern across different CRM and TF

**Figure 1:**
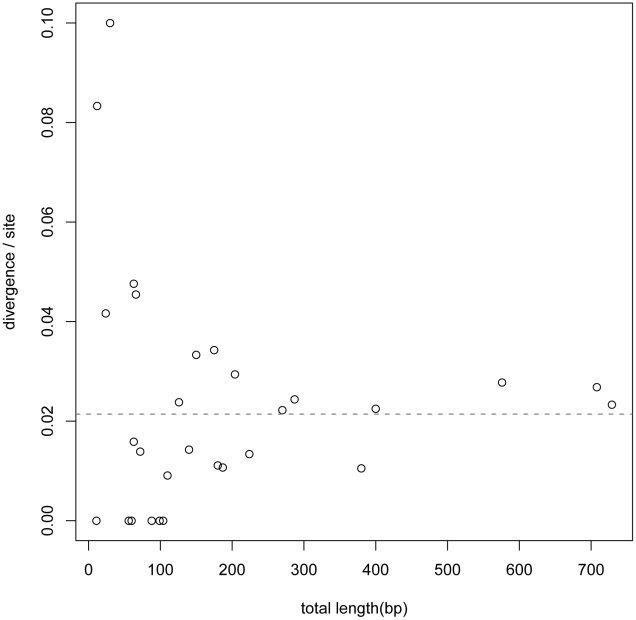
TFBS divergence for 30 TF. TFBS divergence for 30 TFs is plotted as a function of the total number of nucleotides assigned as binding sites to that TF. A maximum likelihood estimate of the mean divergence is marked by the dashed line. Individual binomial tests find no evidence for heterogeneity in divergence rates among the 30 TFs (0.05 significance level, with Bonferroni correction for multiple testing).

Binding sites for an individual TF or a single CRM usually had too few counts of single nucleotide polymorphism or fixed differences to allow informative statistical analysis. Furthermore, the breadth of the turnover phenomenon across almost all investigated TF and CRM suggests a common underlying evolutionary mechanism [5], [7], [8], [18], [31]. We therefore considered pooling observations from across TFs and CRM. To see if the evolutionary rates in different TFs binding sites are sufficiently uniform, we measured sequence divergence between *mel* and *sim* for the 30 TF. After accounting for sample sizes, no significant departure from the average rate is detected by a binomial test (Figure 1). Moreover, the pooling approach should be conservative in deriving a general pattern with respect to among TF variations.

We then estimated percent loss and gain of TFBS on the *mel* and *sim* lineages. For each of the 645 footprint TFBS, a PWM score 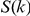 was calculated for each occurrence (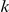) in the alignment of *mel*, *sim* and the inferred *mel-sim* ancestor, by taking the log2 ratio of the probability of a sequence under the functional motif distribution versus that under the genomic background distribution (Material and Methods). Using 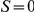 as a cutoff, approximately 2% of all footprint sites were found to be present in *mel* only and may represent *mel* specific gains; and about 2.5% were present in the inferred ancestor (and *mel*) but lost in *sim*. A set of empirical cutoffs were determined for each TF based on the range of PWM scores among its footprint sites, which produced similar results (Table S2). Consistent with the sequence divergence patterns, gain and loss of TFBS appear to be a general pattern across TF and CRM. A total turnover rate of 4.5% between *mel* and *sim* is similar to a previous finding of 5% for a single TF Zeste [5].

We observed approximately equal numbers of gains versus losses in our dataset, although the distribution of these events is asymmetric on the two lineages (16 losses, 0 gain along the *sim* lineage versus 12 gains, 0 losses along the *mel* lineage). This is not unexpected, given that all footprint TFBS were identified as being present in *mel* and the dataset doesn't include *sim*-specific TFBS. We predicted that identification of TFBS by computational methods would produce a more even pattern of gains and losses in both lineages. We tested this prediction for three TF (Hb,Bcd,Kr) using a stringent cutoff procedure and for each TF we found a similar total number of predicted binding sites in the two lineages (Text S1; Figure S1). We thus rejected the (unlikely) possibility that there has been a large-scale evolutionary gain of TFBS in *mel* and loss in *sim*.

### Investigating evolutionary forces for TFBS gain, loss, and maintenance

Gain and loss of TFBS may be subject to distinct evolutionary forces. To investigate them separately, we assigned each mutation within a footprint TFBS in *mel* or *sim* to either affinity-increasing or affinity-decreasing group based on PWM score difference between the ancestral and the derived mutation (Materials and Methods). Bioinformatic and experimental investigation showed that this PWM-based procedure for inferring the direction of binding affinity change is reliable when PWM predicted magnitude of change is not too small (Materials and Methods, Figure S2 and Figure S3). We established a threshold corresponding to a PWM score difference of one, i.e. at least two-fold change in the likelihood ratio between a motif or background distribution, in order to minimize the chance for mis-assignment. Varying this threshold between zero and two do not affect the results qualitatively.

We employed two approaches to investigate evolutionary forces acting on affinity increasing and decreasing changes. One approach is based on contrasting polymorphism and divergence patterns in a McDonald-Kreitman (MK) test framework [32]. Positive selection is expected to inflate substitution relative to polymorphism while negative selection will have the reverse but weaker effect [33]. We used synonymous changes in the target genes for the CRM as a proxy for a neutrally evolving class. Following established practices, we further classified each synonymous change as according to its expected impact on codon bias – No-Change, Preferred-to-Unpreferred, or Unpreferred-to-Preferred – and used the No-Change class as the neutral reference. The second approach investigates the site frequency spectrum of TFBS polymorphism to make inferences about selective pressures acting more recently on binding sites.

### TFBS ascertainment

The fact that all footprints were identified in *mel* impacts the analysis in two ways. First, gains of TFBS can be observed in *mel* but not losses, while the reverse is true in *sim*. Therefore, even though similar processes are most likely operating in both species, our evolutionary analysis of binding site gain will focus on changes in the *mel* lineage, whereas losses will be restricted to changes in the *sim* lineage.

Second, affinity-decreasing and affinity-increasing mutations have the potential to differ in detectability as a footprint site in *mel*. This arises because mutations in TFBS were sampled conditioned on the TFBS being detected in *mel* and affinity-changing mutations in *mel*, in turn, have the potential to affect the detectability of the TFBS. Depending on whether the derived mutation is affinity-increasing or affinity-decreasing, two distinct biases are introduced in the expected neutral frequency spectrum (Figure S4). Given that the dataset consists only of TFBS that are detectable by footprinting, we assume that the high-affinity allele will always be detectable. Consider the possible situation in which the low-affinity allele is not detectable as a footprint: if the derived mutation is affinity-decreasing, the probability of detecting the TFBS will change inversely with the mutant allele frequency; conversely, if the derived mutation is affinity-increasing, the probability of detection will increase with the mutant allele frequency. Substitutions may be viewed as a special instance of a segregating mutation and treated similarly.

This effect of ascertainment on neutral expectations for the MK test and the site frequency spectrum can be modeled analytically (Text S2); there is no ascertainment if both alleles are equally detectable as footprints. To incorporate uncertainty in the detectability of the low-affinity allele, the model incorporates a parameter, *f*, which specifies the probability that the weaker affinity allele will not be detected in the footprint assay. While *f* is likely to be greater than 0, it is unlikely to be close to 1 because footprint sites are degenerate and span a range of affinities. Under the conservative assumption that the lowest affinity among the footprint sites is the detection limit, we estimate 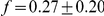 for the 30 TF (Text S2), indicating that the majority of TFBS changes will be detectable.

In the following sections, we first present our analysis of polymorphism and divergence in *mel*, focusing on the forces acting to either maintain functional TFBS or to create new ones. We then turn to *sim*, focusing on TFBS loss. Finally, we analyze the spacer sequences between TFBS in both species.

### Analysis in *mel* suggests potentially positive selection for TFBS gain and purifying selection in maintaining existing TFBS

**Figure 2:**
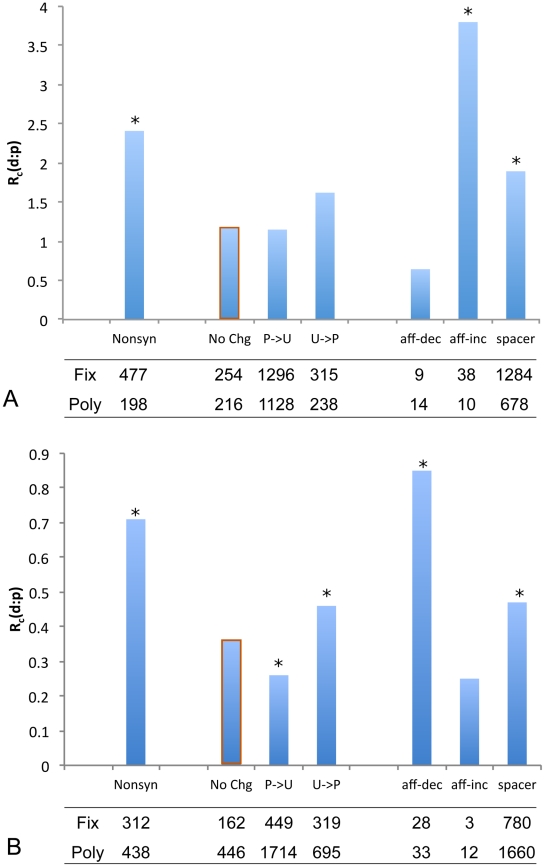
Substitution-to-polymorphism ratios in *mel* and *sim*. 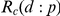
 ratios between number of fixed mutations (fix) in each class and number of common polymorphisms (poly; with derived allele frequency 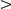
0.15; see text for justification) for (A) *mel* and (B) *sim*. In *sim*, only TFBS with a predicted ancestral PWM score 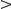
2 are included (see text). Synonymous changes are categorized according the predicted effect of a mutation on codon preference (P: Preferred codon; U: Unpreferred codon; No Chg: P
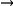
P and U
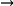
U). Consistent with previous reports, we find evidence for selection on biased codon usage in *sim* but not *mel*. Statistical significance of each class relative to the neutral reference (the No-Change class, outlined in orange) is evaluated by Fishers exact test. Classes that are significant at a 0.05 level (two-sided test) are marked with an asterisk above the bar.

For each class of change we summarized the data in the MK table by calculating the ratio, 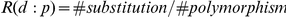. The presence of weakly deleterious mutations can mask signatures of positive selection, and if removed can improve the power of the test [34]. Since most deleterious mutations will be at low frequencies, using 15% as a frequency cutoff has been shown to achieve most of the benefits of a more sophisticated model incorporating the distribution of deleterious effects [35]. We applied this cutoff and denote the ratio of substitutions to common polymorphism by 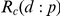. Under this procedure, 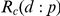 is significantly higher for nonsynonymous changes than for the synonymous No-Change class (Figure 2A), consistent with previous findings of positive selection driving amino acid substitutions in *Drosophila* [36].

**Figure 3:**
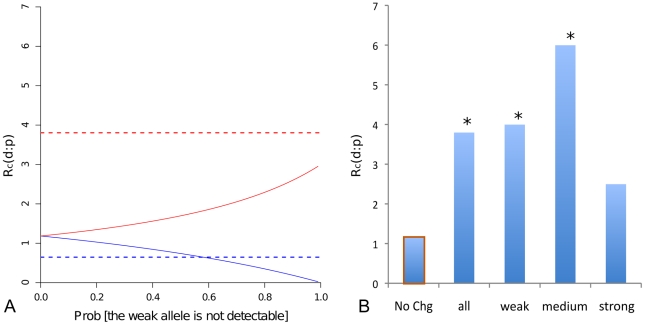
Substitution-to-polymorphism ratio for affinity-increasing mutations in *mel* suggests positive selection. (A) The expected neutral 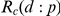 ratio under ascertainment (solid line) as a function of the probability that the weaker allele will not be detectable as a footprint for affinity-decreasing (blue) and affinity-increasing (red) mutations. Dashed lines represent the observed ratios for the two classes respectively. (B) Observed 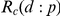 for affinity-increasing mutations within TFBS grouped by predicted ancestral PWM score, compared to the No-Change class (orange box). An asterisk above the bar indicates statistical significance at a 0.05 level by Fishers exact test.

To delineate the effect of ascertainment from that of selection for the affinity-increasing and affinity-decreasing mutations, we compared the observed 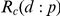 to the expected neutral ratios under the ascertainment with different 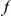 values (Text S2). For affinity-decreasing mutations in *mel*, the difference from the synonymous No-Change class is not statistically significant, even in the absence of ascertainment bias (Figure 3A green, Figure 2A). This seems to suggest only neutral or deleterious mutations are present for this class and therefore no positive selection is involved. The validity of this conclusion can be questioned, however, because any affinity decreasing substitutions in *mel* that led to the loss of a site will not be included in the data while our correction for the ascertainment only accounts for neutral changes but not a potential adaptive excess. Thus, rejection of the neutral model in favor of positive selection is not possible for affinity-decreasing mutations in the *mel* lineage. However, this test is possible for the *sim* lineage (reported in the next section), where the loss of a TFBS is observable.

For affinity-increasing mutations no amount of ascertainment under our model can account for the observed relative excess of substitutions (Figure 3A red). We further reasoned that the ascertainment effect should be weaker or non-existent for TFBS with an ancestrally strong binding affinity, which would be identified with or without the affinity-increasing mutations. We therefore investigated whether the excess of affinity-increasing substitutions differed if TFBS changes were grouped according to the strength of the inferred ancestral binding affinity. We found a consistently larger 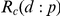 ratio, i.e. an excess of substitutions, across the entire range of inferred ancestral binding affinity classes compared to the No-Change class, including binding sites with the strongest ancestral binding affinity (Figure 3B). These results collectively suggested that positive selection has contributed to the fixation of affinity-increasing changes.

**Figure 4:**
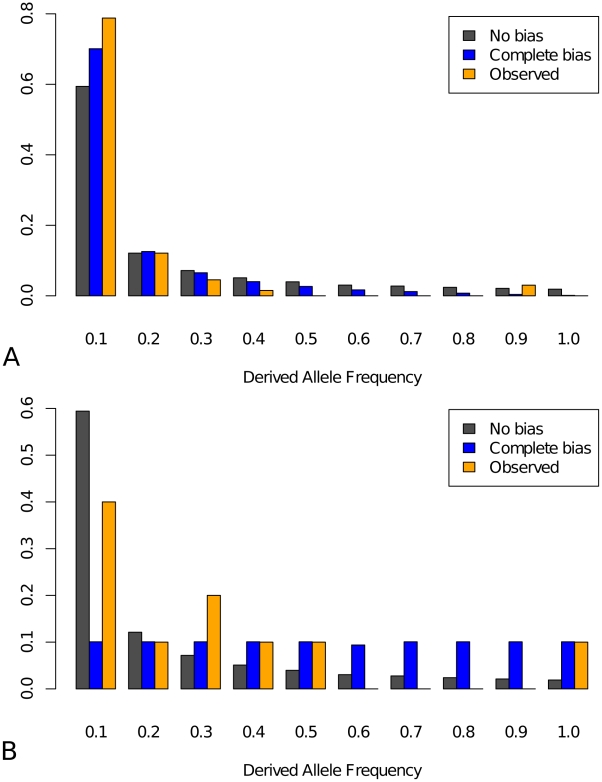
Site frequency spectra. (A) Affinity-decreasing mutations and (B) affinity-increasing mutations. Grey: neutral expectation with no ascertainment (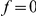); Blue: neutral expectation under complete ascertainment (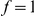); Orange: observed frequency spectrum. The calculations of the expected frequency spectrum under no bias and complete bias are described in supplementary methods. The total number of segregating sites for affinity-decreasing and affinity-increasing mutations is 64 and 15, respectively.

To further investigate evolutionary forces acting on the segregating mutations in TFBS in the population, we utilized the site frequency spectrum, for which we generated the neutral expectations for affinity-increasing and affinity-decreasing mutations separately under ascertainment, with 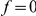 or 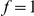 (corresponding to no bias or complete bias, respectively). For affinity-decreasing mutations, with the ascertainment expected to shift the frequency spectrum to lower frequency classes (Figure 4A, blue versus grey bar), the observed spectrum is shifted in that direction but is even more extremely so than the complete bias expectation (Figure 4A, orange versus blue). Since 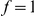 is clearly an overestimate (compared to our estimate of 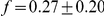), this strongly suggests that forces other than ascertainment must have shaped this pattern. Both a recent selective sweep and population growth can produce an excess of rare variants and one or both mechanisms may be acting in this system, as is suggested by our finding that synonymous changes also show a relative excess of low frequency mutations (Figure S5B). However, as we compared the site frequency spectrum of the affinity-decreasing mutations to that of synonymous sites (corrected for ascertainment), we found the former is again more significantly shifted than the latter (Figure S6). Thus we suggest that the observed frequency spectrum is consistent with on-going purifying selection against affinity decrease in functional TFBS. The observed frequency spectrum for affinity-increasing mutations lies between the two expectations and the differences are not significant from either one, a possible consequence of the small sample size (15 observed affinity-increasing polymorphisms) (Figure 4B). Thus, while positive selection is indicated on the basis of the MK test, inference cannot be made about on-going selection for affinity-increasing mutations.

### Analysis in *sim* suggests loss of TFBS may be adaptive

Patterns of polymorphism and divergence in *sim* are not influenced by the ascertainment because the identification of TFBS in *mel* is independent of the effect of mutations fixed or segregating in *sim*. However, the inclusion of binding sites gained in *mel* may confound the analysis as their orthologous sequences in *sim* may have evolved under less or different kinds of selective constraints. We thus restricted the analysis to footprint TFBS predicted to be present in the *mel-sim* common ancestor, where we found a significant excess of substitutions for the affinity-decreasing mutations compared to the synonymous No-Change class (Figure 2B, Fisher's Exact Test 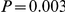). Statistical significance of this pattern is robust to the cutoff for excluding binding sites gained in *mel* (Table S3). A relative excess of substitutions might also be a consequence of factors other than selection, such as systematic differences in the genealogical histories of CRM versus synonymous sites. However, these factors seem unlikely to be the cause of this type of departure from neutrality in these two species (Kohn and Wu 2004). Therefore we consider positive selection a more plausible explanation.

We also compared the ratio between affinity-decreasing and affinity-increasing mutations in polymorphism to the expected ratio of the two classes in the mutational input, i.e. the probability for a new mutation to be one of the two classes (Materials and Methods). Briefly, the expected ratio was obtained by considering all possible mutations in each of the 645 footprint TFBS and their predicted effects on binding affinity the same way as we did before. Assuming polymorphism for both classes were neutral, we expected similar ratios, whereas the observed results showed a significant deficit of affinity-decreasing polymorphism relative to affinity-increasing polymorphism (Table 1), which may suggest that among new mutations, affinity-decreasing ones are more likely to be deleterious, a result consistent with our finding based on frequency spectrum in *mel*. A similar approach has been applied before, using the sum of 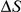 (individual mutation's effect on binding affinity predicted by PWM) within a CRM instead of counts of mutations in binary classes [29]. There the author also found evidence for purifying selection against affinity-decreasing mutations. The finding of both on-going purifying selection and potentially positive selection acting is not dissimilar to patterns found in nonsynonymous changes [36]. We reserve for the Discussion section the attempt to reconcile the adaptive loss of TFBS, as observed between the two species, with on-going purifying selection against affinity-decreasing new mutations.

**Table 1:** Mutational probability of affinity increase and affinity decrease.

Affinity-Class	Mutational Probability	Observed#	Expected	Chisq p-value*
Affinity-increase	0.105	12	4.7	
Affinity-decrease	0.895	33	40.3	0.002

# number of segregating mutations of each class among the 6 *sim* lines;

*chi-square test p-value is based on 10,000 simulations as one of the cells contain less than 5 counts.

### Spacer sequences might contain large numbers of unidentified functional elements

In both *mel* and *sim* we found a significant excess of substitutions in spacer sequences, indicative of positive selection in these intervals (Figure 2). Also, the frequency spectrum for this class is strongly shifted towards lower frequencies (Figure S5E, Tajima's D = −1.09), indicative of on-going purifying selection. The implication of these results is that spacer sequences might contain many unidentified functional elements, for example, TFBS for known or uncharacterized transcription factors, or perhaps other structural features not yet understood.

To summarize, analysis of TFBS changes in *mel* indicates on-going purifying selection against affinity-decreasing polymorphism in the population, and positive selection for affinity-increasing substitutions. In *sim*, the analysis of affinity-decreasing changes indicates a significant, and potentially adaptive excess of substitutions that contributes to binding site loss. Spacer sequences between footprint TFBS in these well-characterized CRM also exhibit patterns of polymorphism and divergence consistent with both functional constraint and adaptive evolution.

## Discussion

Natural selection, both positive and negative, has been shown to act throughout noncoding regions of the Drosophila genome [21], [22], albeit with varying intensities [23]. Against this backdrop of ubiquitous selection in noncoding DNA, should it be surprising to find signatures of positive selection in *Drosophila* TFBS? We think not. More surprising perhaps is the incompatibility of this finding with the model of neutral compensatory binding site turnover, a simple and appealing mechanism that allows for both rapid binding site turnover and functional stasis of CRM activity. But as explained below, there are good reasons to doubt whether a strictly neutral compensatory process can actually generate rapid TFBS turnover in *Drosophila*, even with its favorably large population size. Positive selection, in contrast, can drive arbitrarily fast rates of binding site turnover; the question is whether it can also allow for functional stasis of CRM activity. Below, we first discuss the strengths and limits of our analysis and then we describe properties of gene regulatory networks that can promote adaptive binding site turnover and yet also constrain the function of CRM.

One challenge in investigating cis-evolution is the proper alignment of noncoding sequences. To minimize this potential problem, we specifically selected a pair of closely related sibling species, *D. melanogaster* and *D. simulans* for investigation. Sequence divergence between the two species in noncoding regions ranges only between 5% and 8% [37], which allowed us to accurately identify single nucleotide differences from unambiguous alignments of binding sites (those with alignment gaps were excluded from the analysis). Working with closely related sequences also provided accurate inference of ancestral states, and thus the direction of mutational change along the phylogeny, as well as minimized *trans-cis* co-evolution. Independently, Bradley *et al* also recommended *mel* and *sim* for measuring binding site divergence based on these same issues arising in their analysis of divergence between two more distantly related species [31].

Another challenge in studying TFBS turnover is the establishment of a TFBS dataset consisting of biologically functional sites, a difficult task due to both the high false positive rate in binding site prediction (even in ChIP bound regions) and the difficulty in validating the biological functionality of individual binding sites. While many genome-wide datasets for TFBS are becoming available, several properties of the *Drosophila* DNase I footprint dataset made it the one of choice for use in this study. First, the *in vitro* footprint experiments were applied not to anonymous noncoding regions but rather to specific sequences that had been identified with *in vivo* reporter assays as containing a CRM. Furthermore, the transcription factors assayed for each CRM were also chosen based on prior genetic evidence for their involvement in the regulation of the CRM. For both of these reasons, subsequent experimental analysis of *Drosophila* footprint sites has invariably validated their functionality [38]–[43]. This experimental sampling of footprint site functionality is unique among available TFBS datasets, and provides evidence for a low false positive rate. In contrast, a recent attempt to combine known CRM, ChIP bound regions, and PWM prediction to obtain a genome-wide TFBS dataset estimated 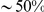 false positive rate [4]. Although the footprint sites were identified in lab strains particular to each individual experiment, we provided reasonings and evidence why the annotation is applicable to natural populations (Text S3). In particular, we constructed phylogenetic trees based on the genomic sequences containing the CRM we studied for natural population lines as well as a representative lab strain (the genome sequence reference strain), which shows that the later is indistinguishable from the rest (Figure S8). This also suggests the lab strains were not genetically divergent from the natural population.

Genome-wide studies have identified signals of both positive and negative selection in noncoding sequences in *Drosophila*, but not the biological or functional basis for this selection. In this study, we distinguished mutations in the footprint sites by their functional impact – either increase or decrease the binding affinity of the corresponding TF – and observed different patterns of polymorphism and divergence between the two classes. For example, we found that affinity-decreasing mutations are on average more deleterious among new mutations than affinity-increasing ones, as revealed by a comparison of the ratio between the two classes in polymorphism with the expectation from mutational input. Such distinctions were not observed when mutations were grouped in other ways irrelevant to the function of TFBS (for example, mutations in the first half of the motif versus the second half). For these reasons we think the evidence supports our specific model of selection acting on binding site gain and loss as opposed to an unidentified functionality in noncoding sequences in general. The mechanism of selection we described here for well-annotated TFBS could in principle be acting more broadly across noncoding regions inasmuch as noncoding DNA is often associated with proteins binding.

Our ability to correctly categorize mutations into affinity-increasing or affinity-decreasing categories hinges on the accuracy of PWM predicted affinity differences. To investigate this issue, we employed a state-of-the-art microfluidics technique, MITOMI [44], to experimentally measure the binding affinity differences for naturally occurring mutations in *hunchback* and *bcd* binding sites. To our knowledge, this is the first time that accurate measurements have been made on population-level variants in TFBS. We found that PWM scores correctly predicted the measured direction of affinity change for 21/25 mutations investigated. Of the four mutations that PWM predicted the wrong direction, three have effect sizes predicted to be close to zero. The PWM-based procedure, therefore, may not be accurate for small predicted differences in binding affinity. Taking these results into consideration, we employed a binary classification of mutations with PWM differences exceeding a threshold requirement rather than using quantitative predictions of all PWM score differences as a basis for our analysis.

Another potential issue concerns applying *mel* derived PWM to score *sim* TFBS binding affinity. Transcription factor protein evolution between the two species, if it occurred, could lead to underestimation of binding affinity in *sim*, although the effect should be similarly applied to both substitutions and polymorphism and thus is not expected to cause a relative excess of the former as observed in the *sim* data. Nevertheless, we show two lines of arguments that suggest this is not the case in our study: first, for the 30 TF whose binding sites we investigated, the DNA bindings domains and other functionally annotated domains are completely conserved except for one biochemically conservative amino acid difference (Asp/Glu) in *Dorsal*s RHD domain (Table S4). Although differences exist in other parts of the proteins, it has been shown that DNA binding domain may singly determine the sequence specificity of the protein [44], [45]. Second, if what we identified as affinity-decreasing mutations in *sim* reflected on-going adaptations to a slightly different motif, we would expect, but did not find, a consistent pattern in the position and kind of nucleotide changes for a TF (data not shown). To further support this argument, we derived PWM using MEME from the *mel* footprint sites as well as their aligned sequences in *sim*. As shown in Figure S7, our classification of binding site differences did not differ between using either the *mel* PWM or the *sim* PWM, contrary to what would be expected if TF sequence specificity had evolved between the two species. Therefore we consider it very unlikely for the 30 TF included in this study to have undergone significant evolution in their sequence specificity. In addition, because the SELEX derived PWM produce consistent results with the footprint derived ones (Figure S3), we can also rule out the possibility of over-optimization of the PWM inducing a sequence preference for *mel* over *sim*.

Finally, in the course of the analysis, we identified and modeled an ascertainment bias caused by the identification of footprint sites exclusively in a single strain of *mel*, and the possibility that sequence changes in the same species can lead to creation or destruction of the footprint feature (as described in the Results section). Many other genomic features such as miRNA binding sites and recombination hotspots can also satisfy these two criteria. As new studies attempt comparative evolutionary studies of genomic features often identified in a single reference sequence, we expect this problem to become more common and, therefore, to require greater attention. If not properly accounted for, this form of ascertainment can lead to false rejection of the neutral hypothesis. The analytical model of ascertainment under neutrality we developed here should be applicable to population genetic and evolutionary analysis of many different structural features of genomes.

Our population genetics analysis identified three major forces in TFBS evolution. First, we found functional TFBS were selectively maintained in the population by purifying selection, as revealed by a frequency spectrum skewed towards rare variants for affinity-decreasing polymorphism in *mel* and a significantly reduced proportion of affinity-decreasing polymorphism compared to mutational input in *sim*. These results are consistent with previous findings of selective constraints on functional TFBS. Mustonen and Lässig estimated that the average selection coefficient to maintain TFBS in bacteria and yeast genomes are on the order of 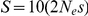 [28], [46], and a similar estimate has been obtained for *Drosophila* [4]. The substitution rate with 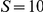 is expected to be less than 0.05% of the neutral rate in a population with a size as large as *Drosophila* (Equation B6.4.1, [47]). This means TFBS loss is unlikely to happen through fixation of deleterious mutations (0.2 losses expected for 645 footprint TFBS versus 16 inferred in *sim*). We can think of only three mechanisms by which TFBS loss can occur at an appreciable rate: (1) there is loss of constraint; (2) a pair of tightly linked compensatory mutations creates an effectively neutral allele; or 3) positive selection drives the loss of TFBS. Our second finding – a significant excess of substitutions compared to the neutral class for affinity-decreasing mutations in *sim* – is consistent only with positive selection for TFBS loss. Occasional adaptive loss of a TFBS is not inconsistent with more ubiquitous selection to maintain binding sites [28], and has been suggested to account for the evolution of fermentation pathways in yeast [16].

Our third finding is positive selection contributing to the gain of TFBS, as revealed by a significant excess of substitutions for affinity-increasing mutations in *mel*. Collectively, the three findings indicate that natural selection is extensively involved in the maintenance, gain, and loss of TFBS. This conclusion challenges the prevailing view of a neutral TFBS turnover process [4], [13].

We think that a selectionist interpretation of the turnover process is plausible for several reasons. First, the assumption of CRM functional stasis, which is the main argument for the neutral (i.e., compensatory) view, is not well supported experimentally. Reporter transgene assays, in particular, are limited in their quantitative resolution, and yet even in these studies, repeatable differences were found between orthologous CRM [7]. A functional rescue experiment is potentially more sensitive than a reporter transgene assay. As applied to the *Drosophila even-skipped* stripe 2 enhancer, it demonstrated clear functional differences between CRM that were previously believed to have the same spatial pattern of expression [48].

Second, compensatory neutral evolution cannot account for the patterns of variation observed in this study. According to this model, affinity-decreasing mutations should in general be deleterious but occasionally become “effectively” neutral when a second compensatory mutation occurs in the CRM of the mutant allele. A mixture of deleterious and compensatory mutations, even if the latter is common, may bring patterns of polymorphism and divergence close to a neutral scenario, but cannot produce a signature of positive selection as observed for both classes of mutations in our analysis. In addition, analytical modeling of the compensatory evolution of TFBS finds that the waiting time for a turnover event is long if complete neutrality of the compensating mutations is assumed [15]. To shorten the waiting time to be compatible with the *Drosophila* TFBS turnover rate, the parameterization of the model requires that the double mutant allele have higher fitness than the non-mutant allele, making it a directional selection model. This supercompensatory scenario could produce signatures of positive selection both for binding site gain and loss, the latter occurring because the fixation of a deleterious mutation in an existing TFBS will have the appearance of being positively selected as it hitchhikes to fixation on the selectively favored allele. However, this scenario is biologically unrealistic, as it requires the second mutation (the gain of a TFBS) to be positively selected only on the background of the first mutation.

As an alternative, consider the following model of positive selection on CRM structure/function. We propose that for CRM with large numbers of interacting partners, the network of cis- and trans-factors will inevitably be constantly evolving – due to both direct selective pressures imposed on the CRM or indirect effects caused by adaptations in other components of the network. For example, egg length variations between and within *Drosophila* species have been studied as potentially adaptive traits; if egg length evolves, genes such as *eve* whose expression pattern need to scale with the embryo may need to change its CRM to adapt to the new context [49]. This constant flux of change, we propose, imposes continual selection pressure for CRM function within the network to co-evolve and change. This “moving target” hypothesis finds support in an analytical study, which shows that fluctuating selection may be common in *Drosophila*, with changes in the sign of selection coefficient occurring at nearly the rate of neutral evolution [50]. Adaptive substitutions could therefore occur before selection switches its sign again, since positively selected mutations fix at rates much higher than the neutral mutation rate.

**Figure 5:**
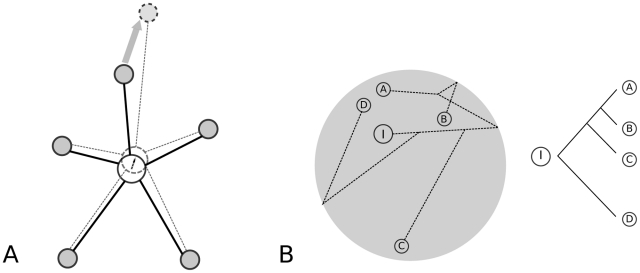
Models of CRM evolution with changes in fitness optimum. (A) The central node represents the CRM of interest and is connected to many interacting partners. With increasing number of connecting partners, we expect the CRM function to change more frequently in small steps but at the same time to be more constrained in function space. (B) A hypothetical evolutionary trajectory in CRM function space. Small changes in a system under global constraints result in non-linear functional evolution with time. The circle represents permissible space within which CRM function can change without causing strong pleiotropic effects. Depicted on the right is the species phylogeny. Starting from I, the ancestor of the existing species, the CRM function moves in the constrained region and generates a non-clock like evolution pattern in the extant species–species A and D are most distantly related phylogenetically but most similar functionally.

At the same time, the high connectivity in the regulatory network implies pleiotropic effects while the essentiality of genes controlled by the network may call for accurate regulation, both suggesting that the net change in CRM function will be highly constrained (Figure 5A). Under this conceptual model, functionally significant change will be possible on short evolutionary timescales, but will remain within constrained bounds over longer timescales. This feature of the model would account for adaptive gain and loss of TFBS in CRM, and could explain the strongly non-linear relationship between function and sequence evolution as exemplified by the *Drosophila eve* stripe 2 enhancer [7], [8]. Moreover, it provides an explanation for the finding of a non-clocklike evolutionary pattern: sequences from *D. pseudoobscura* rescues a *mel eve* stripe 2 enhancer deficiency almost as well as the native *mel* enhancer and substantially better than ones from much more closely related species ([48], Figure 5B).

In conclusion, our findings provide empirical evidence for positive natural selection acting in CRM and TFBS evolution. We suggest that CRM are not as functionally static as commonly believed, but rather may experience frequent adaptation through binding site turnover, even though there may be constraints on net change over longer evolutionary time.

## Materials and Methods

### CRM annotation and sequence alignments

REDfly [30] is a database of manually curated CRM and TFBS obtained from the literature from which we chose 118 non-overlapping autosomal CRM for investigation (Table S1). They regulate 81 target genes and contain binding sites for 82 TF. The 118 CRM range in size from 65 bp to 4.3 kb (median = 515 bp) and contain between 1 to 64 DNase I footprint sites (median = 4). From the set of 82 TF, we identified a subset of 30 with more than 10 footprint sites represented in the dataset and with carefully constructed Position Weight Matrices [51]. In each footprint region plus five flanking bases on each end, we applied the appropriate position weight matrix to identify the highest scoring match as the core motif for the TFBS (referred to as TFBS in the text). We only included those TFBS for which the alignment between *mel* and *sim* sequences contain no insertions or deletions (including both fixed or polymorphic sites). As a result, a total of 645 TFBS for these 30 TF were included for analysis.

For each of the 118 CRM (coordinates in dm3 of *D. melanogaster* reference genome listed in Table S1), we downloaded pre-aligned MAF blocks from UCSC genome browser for *D. melanogaster* (*mel*), *D. simulans* (*sim*), *D. sechellia* (*sec*), and two outgroup species, *D. yakuba* (*yak*) and *D. erecta* (*ere*). *D. sechellia* is a sister species to *D. simulans* and is included to compensate for the low sequence completeness in the reference *sim* genome. We then used the baseml module in PAML 4.4c [52] to reconstruct the ancestral sequences from the alignments. Following analysis involving polarized changes were done either using a single ancestral sequence for *mel* and *sim* determined by the most probable ancestral state (A,C,G or T) at each position, or summing over the posterior probabilities of all four possible states (full Bayesian approach). The two methods produced essentially the same results and therefore we only presented results using the most probable ancestral state. A maximum parsimony method was also investigated and was found to produce consistent results.

For polymorphism analysis, alignments for the same 118 CRM regions were obtained of a population sample of 162 *D. melanogaster* lines (http://www.hgsc.bcm.tmc.edu/projects/dgrp/) and six *D. simulans* lines (http://www.dpgp.org/). We also compiled the genome sequences of 150 coding regions corresponding to the target genes of the CRM listed in REDfly, for the purpose of compiling synonymous and nonsynonymous changes. For these data, we used codeml module in PAML 4.4c to reconstruct the ancestral sequence states following otherwise the same procedure as described above for CRM regions.

### Position Weight Matrix (PWM)

PWM for 30 TF (Antp, Deaf1, Dfd, Kr, Mad, Trl, Ubx, Abd-A, Ap, Bcd, Br-Z1, Br-Z2, Br-Z3, Brk, Cad, Dl, En, Eve, Hb, Kni, Ovo, Pan, Prd, Slbo, Tin, Tll, Twi, Vvl, Z, Zen) were obtained from [51]. This set represents all the TF for which Down *et al.* identified a single best motif for the REDfly footprint sites. For comparison, we also constructed five PWM (Hb, Bcd, Kr, Prd, Twi) from SELEX (Systematic Evolution of Ligands by EXponential enrichment) data (kindly provided by Mark Biggin). We ran MEME [53] with parameters “-evt 0.01 -dna -nmotifs 3 -minw A -maxw B -nostatus -mod zoops -revcomp text” on different selection rounds of the SELEX data. The best PWM was chosen based on the MEME score, percentage of footprint sites recovered and a penalty for the number of additional matches predicted in addition to the footprint sites (Table S5).

### Use PWM to predict mutation effect on binding affinity

Consider a mutation at the 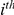 position in a binding site motif involving a change from nucleotide 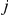 to 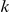 (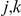 take values 1–4, corresponding to the nucleotides ACGT). We calculated 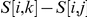, where 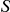 is the PWM matrix of size 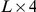. According to previous theories, the PWM score is proportional to the physical discrimination energy of the protein to the sequence and therefore the above calculation may be used to infer the direction and magnitude of binding energy change due to a mutation [54].

To evaluate the accuracy of the PWM-based inference, we experimentally measured the binding energy change of observed mutations in Hb binding sites, using a state-of-the-art microfluidics device that has high sensitivity for relatively weak molecular interactions (MITOMI, [44]). The experiments were performed as described in Maerkl *et al.* [44]. Sixty-four oligonucleotides were synthesized to test 25 SNP in Hb footprint sites and their combination in cases of multiple SNPs in a single TFBS between *mel* and *sim*. Data were analyzed in GenePix 6.0, R, and Prism 5.0. We found that the PWM we used correctly predicted the direction of change in 21/25 cases (Figure S2). Three of the four disagreements had a predicted PWM score change 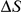 close to or smaller than one, which indicates that PWM may not be accurate when its predicted binding energy differences are small. To minimize the chance of misassigning the direction of binding energy change to a mutation, we set a threshold corresponding to a PWM score difference of one, and classified mutations within (smaller in absolute value) that bound as uncertain. The conclusions are robust to the setpoint of the threshold (for example, Table S3). We also compared the PWM derived by Down *et al.* to the five PWM derived from SELEX data: 97% (33/34) of mutations in the TFBS were consistently classified after excluding nine mutations with small predicted effects by either PWM (Figure S3).

### Rate of gain and loss of TFBS in *mel* and *sim*

To examine the extent of binding sites gain and loss between the two species, we calculated PWM scores 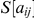 for each of the 645 footprint TFBS (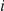 from 1 to 645) in orthologous sequences in *mel*, *sim* or the inferred *mel-sim* ancestor (j from 1 to 3), using patser v3e (by Gerald Z. Hertz, 2002). To determine whether a sequence is a binding site or not, we established two sets of cutoffs for PWM scores. First, we used PWM score 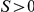, corresponding to the sequence being more likely from a binding site distribution than from a background distribution. For the second we used a set of TF-specific cutoff values chosen by first ranking all footprint sites of a TF by their PWM scores in descending order and then taking the 80% quantile value. The two cutoff set produced similar results (Table S2).

### Construct *sim*-PWM from orthologous sequences to the *mel* footprint sites

To test whether the *mel*-derived PWM might be over-optimized so that they would favor *mel* over *sim* sequences independent of the binding affinity differences, we ran MEME on both *mel* footprint sites for three TF (Hb, Bcd, Trl) and their *sim* orthologous sequences with the same parameters. The two set of ÒorthologousÓ PWM were then applied to score the observed variations in the TFBS of the three TF for comparison (Figure S7).

### Mutational probability for affinity-increasing and affinity-decreasing mutations

We attempted to estimate the probability for a random new mutation to be affinity-increasing (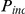) or affinity-decreasing (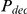) by examining all possible mutations that can occur on the inferred ancestral sequence of mel and sim for the 645 footprint TFBS. At the 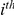 site in a TFBS for TF x, the probabilities are calculated as:
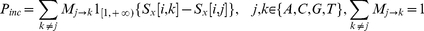
(1)

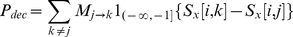
(2)

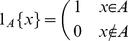
(3)where 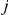 is the original nucleotide and 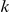 varies among the three possible mutations. 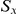 is the position weight matrix for TF x of size 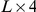. These values were then summed across all 645 TFBS and divided by the total number of nucleotides involved. Mutation matrix 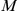 is derived from polymorphism of the 4-fold degenerate sites of 9,628 genes in *D. simulans* [55].

### Generalized McDonald Kreitman (MK) test and site frequency spectrum analysis

For the generalized MK test, we counted the number of fixed and segregating sites for different functional categories in both *mel* and *sim* lineages. In *sim*, we required at least two of the six alleles to be non-missing for a site to be included in the analysis. For coding regions, synonymous sites were further classified into No-Change, Preferred-to-Unpreferred and Unpreferred-to-Preferred, following [22]. Polymorphism and divergence sites in both coding and CRM regions were counted using perl scripts adapted from Polymorphorama (Peter Andolfatto, Doris Bachtrog, 2009).

Following the suggestion of [34], we considered only common polymorphism (derived allele frequency 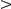
15%) in the generalized MK test to alleviate the problem caused by negatively selected mutations in detecting positive selection. For each mutation category, we compared the substitution-to-polymorphism ratio to the synonymous No-Change class using Fisher's Exact Test. Two-sided p-values are reported.

Site frequency spectrum (*mel* only): Next-generation sequencing data produce variable coverage. To estimate the site frequency spectrum, for each variable site (TFBS, coding and spacers) with a coverage greater than or equal to 150 (maximum is 162) we randomly chose 150 and combined the counts for each frequency class (from 1/150 to 149/150).

## Supporting Information

Figure S1 *De novo* TFBS prediction show potential compensatory sites in *sim* (A), (C) and (E), Proportions of predicted matches to *Hunchback (hb), Bicoid (bcd) or Krpple (Kr)* PWM that are *mel*-specific, *sim*-specific or shared in both species in each BCD or KR regulated enhancer region (defined as regions that contain at least one mel footprint site for the TF). Numbers in the white bar indicate the number of shared predicted sites. (B), (D) and (F) are similar to (A),(C),(E) except that they include 200 bp flanking sequences on each side of an enhancer.
(PDF)

Figure S2 Binding affinity change predicted by *hb* PWM compared to in-vitro direct measurement by MITOMI. MITOMI experiments were performed as described in the methods. Each mutation was measured in two oligonucleotides carrying the original and mutant nucleotide respectively. The two dashed lines indicate the cutoff we applied in the study.
(PNG)

Figure S3 PWM based on mel footprints and SELEX PWM produce consistent results. Each point represents one substitution and its x, y values are the estimates of its effect on binding affinity using the footprint PWM or the SELEX PWM, respectively. 33/34 strong-effect substitutions are consistently assigned by the two sets of PWM into either affinity-increasing or affinity-decreasing categories.
(PDF)

Figure S4 Impact of ascertainment on the detectability of a mutation in *mel*. Each box represents a TFBS, where orange indicates relatively strong binding affinity while greens indicates weak affinity. Each column is an alignment of a sample of six *mel* alleles with the inferred ancestral allele. In the first column, a fixed affinity-decreasing mutation in *mel* with a relatively large effect makes the TFBS not detectable as a footprint. In column 2 and 3, the affinity-decreasing mutations are not fixed but segregating, therefore the probability of not detecting the TFBS is proportional to the derived allele frequency (assuming a random *mel* allele is used in the footprint assay). Column 3–6 illustrate the situation for affinity-increasing mutations, where the substitutions are always detectable but the segregating mutations are detected with higher probability when the derived allele frequency is low. The last two columns represent cases where both alleles are detectable. To incorporate the uncertainty in the detectability of the low-affinity allele, we define a parameter *f* for the probability that the weak allele is not detectable.
(PDF)

Figure S5 Site frequency spectra for different classes compared to the neutral expectation (A) Non-synonymous; (B) Synonymous No-Change (C) Preferred-to-Unpreferred; (D) Unpreferred-to-Preferred; (E) Spacers in CRM. Black: neutral expectation; Gray: observed site frequency spectrum.
(PDF)

Figure S6Relative excess of rare variants suggests purifying selection on affinity decreasing mutations in *mel*. The proportion of low frequency class(es) for affinity-decreasing mutations compared to the theoretical neutral expectation, the observed synonymous sites, or the expected proportion for synonymous sites under ascertainment assuming 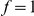. DAF: derived allele frequency.(PDF)

Figure S7 PWM derived from *mel* footprints (PWMmel) or their aligned sequences in *sim* (PWMsim) produce consistent results under our classification method. On the scatter plot each point represents a single nucleotide mutation with its x, y values being the estimates of its effect on binding affinity using either the *mel* PWM or the *sim* PWM, respectively. Green and red triangles are mutations occurring on *mel* or sim lineages. From the figure, the PWM have very little biases with respect to scoring mutations from the species where it is derived or the other species.
(PDF)

Figure S8 A geneaology tree based on 10 kb CRM sequences for 162 lines from DGRP and the Berkeley reference sequencing strain. The tree is built in MEGA using maximum likelihood method, based on 10 kb sequence alignments. It is rooted with one sequence from a closely related species *D. sechellia* as an outgroup (bold and blue). The reference sequencing strain (referred to as lab, bold and red) is obviously inter-mingled with the other 162 lines. A similar procedure on 3 different 10 kb sequences sampled from the genome produced similar shaped trees with the lab line embedded among the 162 lines, although the exact orders of branches are not the same, reflecting different geneaologies between regions in the genome.
(PDF)

Table S1 CRM studied in this study.
(PDF)

Table S2 Percentage of gain and loss of TFBS predicted by two set of cutoffs.
(PDF)

Table S3 The pattern of excess for aff-dec mutations in sim is robust to choices of cutoff.
(PDF)

Table S4 TF protein sequence divergence in different functional regions between *mel* and *sim*.
(PDF)

Table S5 PWM derived from SELEX sequences using MEME.
(PDF)

Text S1 Computational prediction of TFBS in CRM.
(DOC)

Text S2 Neutral expectations for the MK test and the site frequency spectrum under ascertainment.
(DOC)

Text S3 Validity of transferring the footprint sites identified in lab strains to the natural populations.
(DOC)

## References

[1] Schmidt D, Wilson MD, Ballester B, Schwalie PC, Brown GD (2010). Five-Vertebrate ChIP-seq Reveals the Evolutionary Dynamics of
Transcription Factor Binding.. Science.

[2] Balhoff JP, Wray GA (2005). Evolutionary analysis of the well characterized endo16 promoter
reveals substantial variation within functional sites.. Proc Natl Acad Sci U S A.

[3] Dermitzakis ET, Clark AG (2002). Evolution of Transcription Factor Binding Sites in Mammalian Gene
Regulatory Regions: Conservation and Turnover.. Mol Biol Evol.

[4] Kim J, He X, Sinha S (2009). Evolution of Regulatory Sequences in 12 Drosophila
Species.. PLoS Genet.

[5] Moses AM, Pollard DA, Nix DA, Iyer VN, Li XY (2006). Large-scale turnover of functional transcription factor binding
sites in Drosophila.. PLoS Comput Biol.

[6] Gregor T, Mcgregor APP, Wieschaus EFF (2008). Shape and function of the Bicoid morphogen gradient in dipteran
species with different sized embryos.. Dev Biol.

[7] Hare EE, Peterson BK, Iyer VN, Meier R, Eisen MB (2008). Sepsid even-skipped Enhancers Are Functionally Conserved in
Drosophila Despite Lack of Sequence Conservation.. PLoS Genet.

[8] Ludwig MZ, Patel NH, Kreitman M (1998). Functional analysis of eve stripe 2 enhancer evolution in
Drosophila: rules governing conservation and change.. Development (Cambridge, England).

[9] Arnosti DN, Barolo S, Levine M, Small S (1996). The eve stripe 2 enhancer employs multiple modes of
transcriptional synergy.. Development (Cambridge, England).

[10] Shimell MJ, Peterson AJ, Burr J, Simon JA, O'Connor MB (2000). Functional analysis of repressor binding sites in the iab-2
regulatory region of the abdominal-A homeotic gene.. Developmental biology.

[11] Swanson CI, Evans NC, Barolo S (2010). Structural rules and complex regulatory circuitry constrain
expression of a Notch- and EGFR-regulated eye enhancer.. Developmental cell.

[12] Ludwig MZ, Bergman C, Patel NH, Kreitman M (2000). Evidence for stabilizing selection in a eukaryotic enhancer
element.. Nature.

[13] Ludwig MZ, Kreitman M (1995). Evolutionary dynamics of the enhancer region of even-skipped in
Drosophila.. Molecular biology and evolution.

[14] Kimura M (1985). The role of compensatory neutral mutations in molecular
evolution.. Journal of Genetics.

[15] Durrett R, Schmidt D (2008). Waiting for Two Mutations: With Applications to Regulatory
Sequence Evolution and the Limits of Darwinian Evolution.. Genetics.

[16] Ihmels J, Bergmann S, Gerami-Nejad M, Yanai I, McClellan M (2005). Rewiring of the Yeast Transcriptional Network Through the
Evolution of Motif Usage.. Science.

[17] Kuo D, Licon K, Bandyopadhyay S, Chuang R, Luo C (2010). Coevolution within a transcriptional network by compensatory
trans and cis mutations.. Genome research.

[18] McGregor AP, Shaw PJ, Hancock JM, Bopp D, Hediger M (2001). Rapid restructuring of bicoid-dependent hunchback promoters
within and between Dipteran species: implications for molecular
coevolution.. Evol Dev.

[19] Shaw PJ, Wratten NS, McGregor AP, Dover GA (2002). Coevolution in bicoid-dependent promoters and the inception of
regulatory incompatibilities among species of higher
Diptera.. Evolution & development.

[20] Andolfatto P (2008). Controlling type-I error of the McDonald-Kreitman test in
genomewide scans for selection on noncoding DNA.. Genetics.

[21] Andolfatto P (2005). Adaptive evolution of non-coding DNA in
Drosophila.. Nature.

[22] Haddrill PR, Bachtrog D, Andolfatto P (2008). Positive and negative selection on noncoding DNA in Drosophila
simulans.. Molecular biology and evolution.

[23] Kohn MH, Fang S, Wu CI (2004). Inference of Positive and Negative Selection on the 5 â
Regulatory Regions of Drosophila Genes.. Molecular Biology and Evolution.

[24] Torgerson DG, Boyko AR, Hernandez RD, Indap A, Hu X (2009). Evolutionary Processes Acting on Candidate cis-Regulatory Regions
in Humans Inferred from Patterns of Polymorphism and
Divergence.. PLoS Genet.

[25] Bachtrog D (2008). Positive Selection at the Binding Sites of the Male-Specific
Lethal Complex Involved in Dosage Compensation in
Drosophila.. Genetics.

[26] Macdonald SJ, Long AD (2005). Identifying signatures of selection at the enhancer of split
neurogenic gene complex in Drosophila.. Molecular biology and evolution.

[27] Doniger SW, Fay JC (2007). Frequent Gain and Loss of Functional Transcription Factor Binding
Sites.. PLoS Comput Biol.

[28] Mustonen V, Lässig M (2005). Evolutionary population genetics of promoters: predicting binding
sites and functional phylogenies.. Proceedings of the National Academy of Sciences of the United States of
America.

[29] Moses AM (2009). Statistical tests for natural selection on regulatory regions
based on the strength of transcription factor binding sites.. BMC evolutionary biology.

[30] Gallo SM, Gerrard DT, Miner D, Simich M, Des Soye B (2010). REDy v3.0: toward a comprehensive database of transcriptional
regulatory elements in Drosophila.. Nucleic Acids Research.

[31] Bradley RK, Li XY, Trapnell C, Davidson S, Pachter L (2010). Binding Site Turnover Produces Pervasive Quantitative Changes in
Transcription Factor Binding between Closely Related Drosophila
Species.. PLoS Biol.

[32] McDonald JH, Kreitman M (1991). Adaptive protein evolution at the Adh locus in
Drosophila.. Nature.

[33] Sawyer SA, Hartl DL (1992). Population Genetics of Polymorphism and
Divergence.. Genetics.

[34] Fay JC, Wyckoff GJ, Wu CI (2001). Positive and negative selection on the human
genome.. Genetics.

[35] Charlesworth J, Eyre-Walker A (2008). The McDonald-Kreitman Test and Slightly Deleterious
Mutations.. Mol Biol Evol.

[36] Smith NGC, Eyre-Walker A (2002). Adaptive protein evolution in Drosophila.. Nature.

[37] Halligan DL, Keightley PD (2006). Ubiquitous selective constraints in the Drosophila genome
revealed by a genome-wide interspecies comparison.. Genome research.

[38] Arnosti DN, Barolo S, Levine M, Small S (1996). The eve stripe 2 enhancer employs multiple modes of
transcriptional synergy.. Development (Cambridge, England).

[39] Beall EL, Manak JR, Zhou S, Bell M, Lipsick JS (2002). Role for a drosophila myb-containing protein complex in
site-specific dna replication.. Nature.

[40] Chen L, O'Keefe SL, Hodgetts RB (2002). Control of dopa decarboxylase gene expression by the
broad-complex during metamorphosis in drosophila.. Mechanisms of Development.

[41] Lunde K, Trimble JL, Guichard A, Guss KA, Nauber U (2003). Activation of the knirps locus links patterning to morphogenesis
of the second wing vein in drosophila.. Development.

[42] Yan H, Canon J, Banerjee U (2003). A transcriptional chain linking eye specification to terminal
determination of cone cells in the drosophila eye.. Developmental Biology.

[43] Yan SJ, Gu Y, Li WX, Fleming RJ (2004). Multiple signaling pathways and a selector protein sequentially
regulate drosophila wing development.. Development.

[44] Maerkl SJ, Quake SR (2007). A systems approach to measuring the binding energy landscapes of
transcription factors.. Science (New York, NY).

[45] Badis G, Berger MF, Philippakis AA, Talukder S, Gehrke AR (2009). Diversity and complexity in DNA recognition by transcription
factors.. Science (New York, NY).

[46] Mustonen V, Kinney J, Callan CG, Lässig M (2008). Energy-dependent fitness: A quantitative model for the evolution
of yeast transcription factor binding sites.. Proceedings of the National Academy of Sciences.

[47] Charlesworth B, Charlesworth D (2010). Elements of Evolutionary Genetics.

[48] Ludwig MZ, Palsson A, Alekseeva E, Bergman CM, Nathan J (2005). Functional Evolution of a cis-Regulatory Module.. PLoS Biol.

[49] Lott SEE, Kreitman M, Palsson A, Alekseeva E, Ludwig MZZ (2007). Canalization of segmentation and its evolution in
drosophila.. Proc Natl Acad Sci U S A.

[50] Mustonen V, Lässig M (2007). Adaptations to uctuating selection in Drosophila.. Proceedings of the National Academy of Sciences of the United States of
America.

[51] Down TA, Bergman CM, Su J, Hubbard TJP (2007). Large-Scale Discovery of Promoter Motifs in Drosophila
melanogaster.. PLoS Comput Biol.

[52] Yang Z (2007). PAML 4: Phylogenetic Analysis by Maximum
Likelihood.. Molecular Biology and Evolution.

[53] Bailey TL, Williams N, Misleh C, Li WW (2006). MEME: discovering and analyzing DNA and protein sequence
motifs.. Nucleic acids research.

[54] Berg OG, von Hippel PH (1987). Selection of DNA binding sites by regulatory proteins.
Statisticalmechanical theory and application to operators and
promoters.. Journal of molecular biology.

[55] Lu J, Shen Y, Wu Q, Kumar S, He B (2008). The birth and death of microRNA genes in
Drosophila.. Nature Genetics.

