# Supplementary material for: Does Positive Selection Drive Transcription Factor Binding Site Turnover? A Test with Drosophila Cis-Regulatory Modules
Source: PLoS Genet. 2011 Apr 28;7(4):e1002053. doi: 10.1371/journal.pgen.1002053 (PMC3084208; doi:10.1371/journal.pgen.1002053)
Supplement: Figure S1 — De novo TFBS prediction show potential compensatory sites in sim (A), (C) and (E), Proportions of predicted matches to Hunchback (hb), Bicoid (bcd) or Krpple (Kr) PWM that are mel-specific, sim-specific or shared in both species in each BCD or KR regulated enhancer region (defined as regions that contain at least one mel footprint site for the TF). Numbers in the white bar indicate the number of shared predicted sites. (B), (D) and (F) are similar to (A),(C),(E) except that they include 200 bp flanking sequences on each side of an enhancer. (PDF) [file pgen.1002053.s001.pdf]

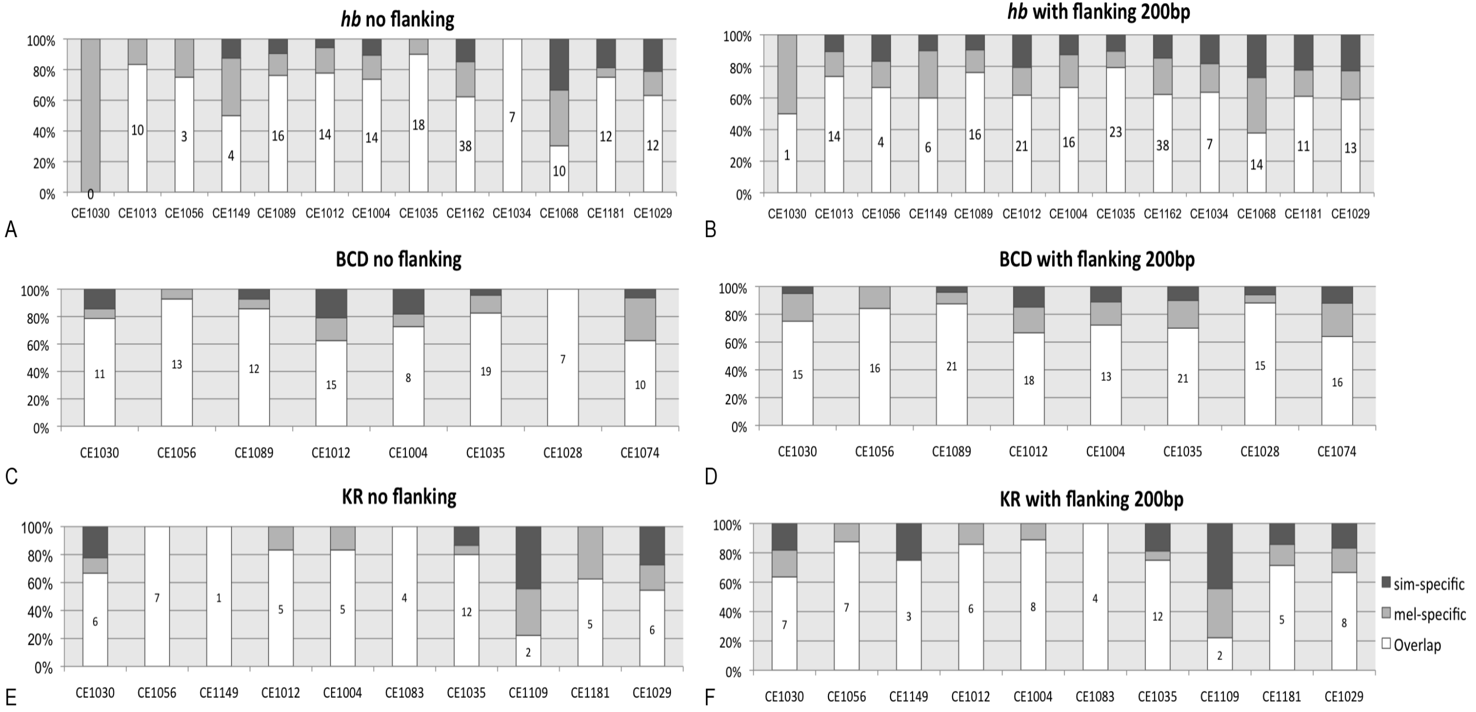
hb no flanking
hb with flanking 200bp
BCD no flanking
BCD with flanking 200bp
KR no flanking
KR with flanking 200bp
100%
80%
60%
40%
20%
0%
CE1030
CE1013
CE1056
CE1149
CE1089
CE1012
CE1004
CE1035
CE1162
CE1034
CE1068
CE1181
CE1029
CE1028
CE1074
CE1083
CE1109
sim-specific
mel-specific
Overlap
A
B
C
D
E
F
