# Supplementary figures and images for: Does Positive Selection Drive Transcription Factor Binding Site Turnover? A Test with Drosophila Cis-Regulatory Modules
Source: PLoS Genet. 2011 Apr 28;7(4):e1002053. doi: 10.1371/journal.pgen.1002053 (PMC3084208; doi:10.1371/journal.pgen.1002053)

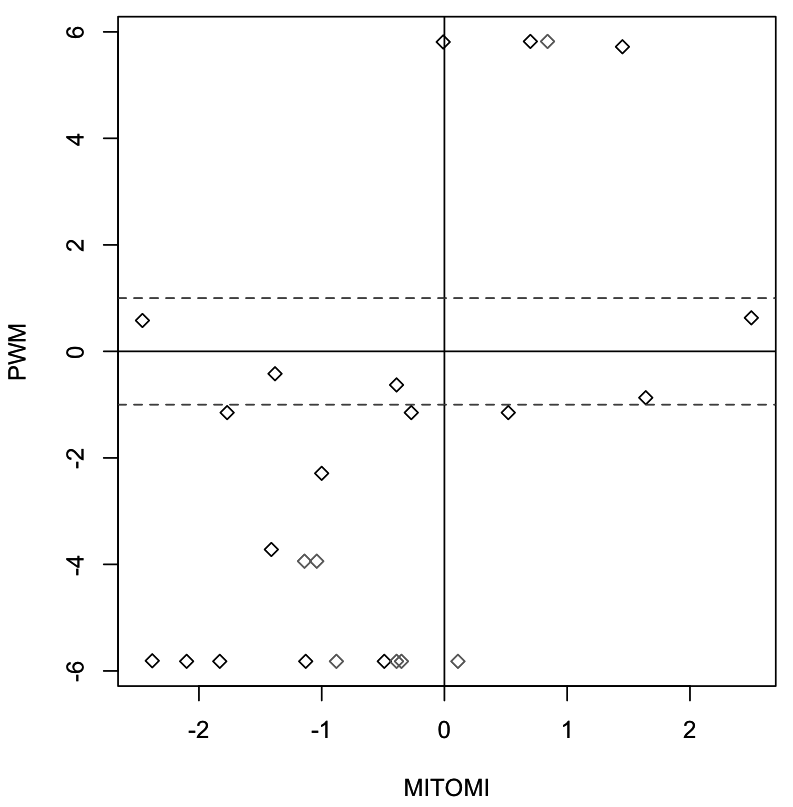

Supplement: Figure S2 — Binding affinity change predicted by hb PWM compared to in-vitro direct measurement by MITOMI. MITOMI experiments were performed as described in the methods. Each mutation was measured in two oligonucleotides carrying the original and mutant nucleotide respectively. The two dashed lines indicate the cutoff we applied in the study. (PNG) [file pgen.1002053.s002.png]

SELEX PWM

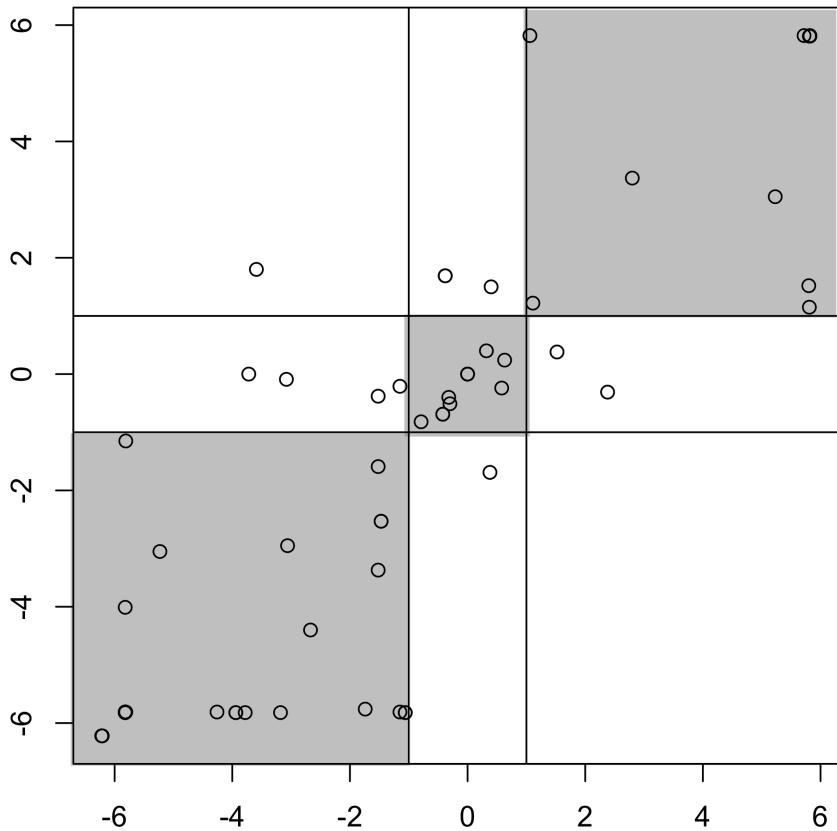

footprint PWM

A

Supplement: Figure S3 — PWM based on mel footprints and SELEX PWM produce consistent results. Each point represents one substitution and its x, y values are the estimates of its effect on binding affinity using the footprint PWM or the SELEX PWM, respectively. 33/34 strong-effect substitutions are consistently assigned by the two sets of PWM into either affinity-increasing or affinity-decreasing categories. (PDF) [file pgen.1002053.s003.pdf]

anc

mel

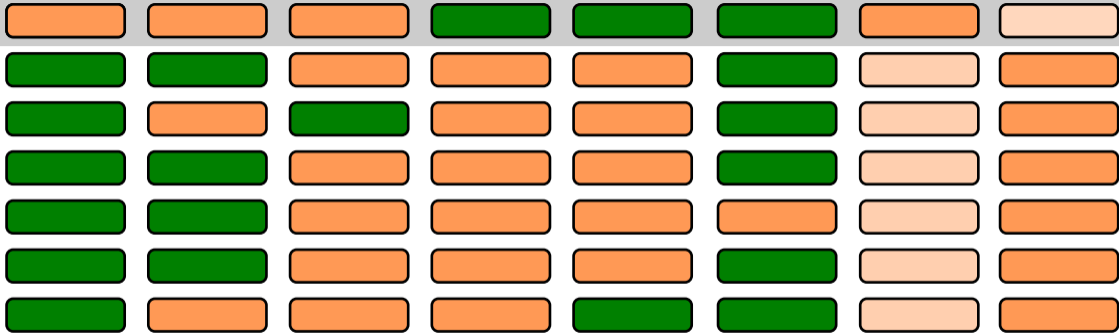

Supplement: Figure S4 — Impact of ascertainment on the detectability of a mutation in mel. Each box represents a TFBS, where orange indicates relatively strong binding affinity while greens indicates weak affinity. Each column is an alignment of a sample of six mel alleles with the inferred ancestral allele. In the first column, a fixed affinity-decreasing mutation in mel with a relatively large effect makes the TFBS not detectable as a footprint. In column 2 and 3, the affinity-decreasing mutations are not fixed but segregating, therefore the probability of not detecting the TFBS is proportional to the derived allele frequency (assuming a random mel allele is used in the footprint assay). Column 3–6 illustrate the situation for affinity-increasing mutations, where the substitutions are always detectable but the segregating mutations are detected with higher probability when the derived allele frequency is low. The last two columns represent cases where both alleles are detectable. To incorporate the uncertainty in the detectability of the low-affinity allele, we define a parameter f for the probability that the weak allele is not detectable. (PDF) [file pgen.1002053.s004.pdf]

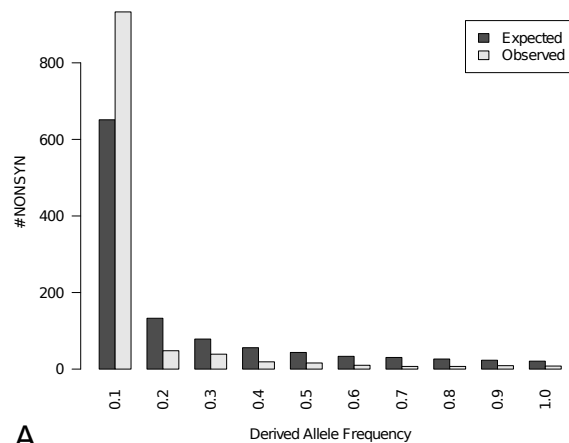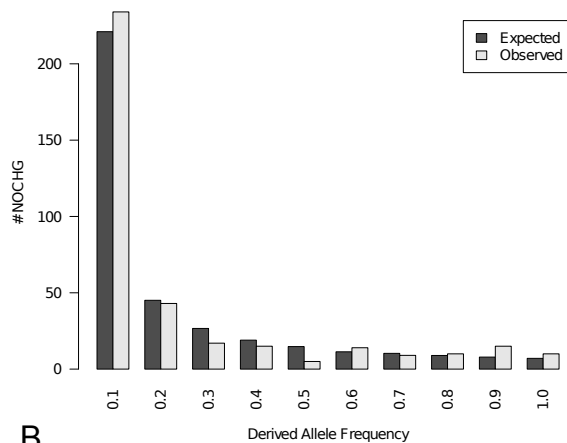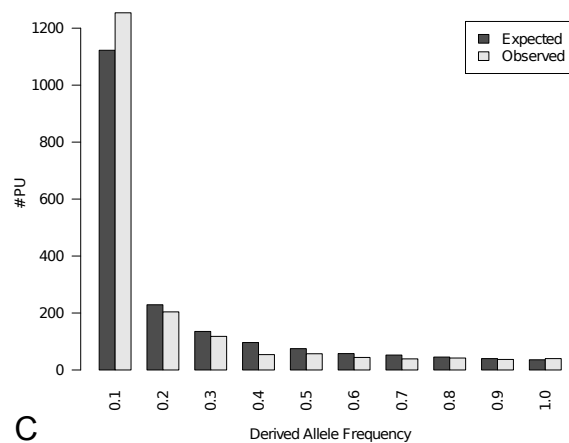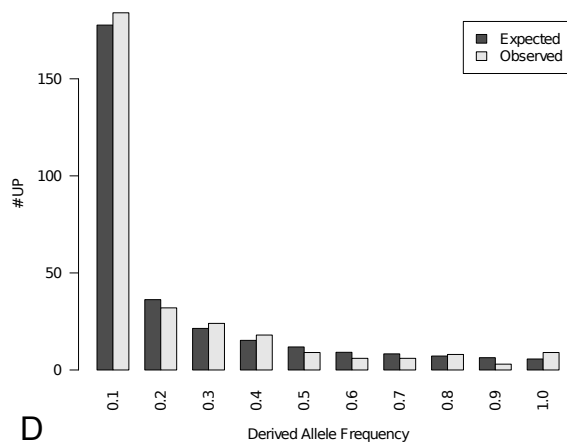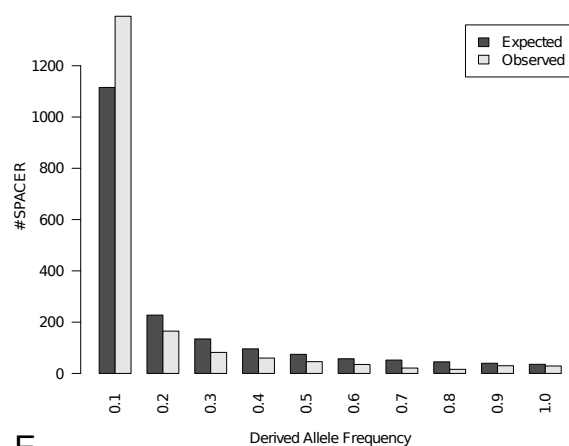

Supplement: Figure S5 — Site frequency spectra for different classes compared to the neutral expectation (A) Non-synonymous; (B) Synonymous No-Change (C) Preferred-to-Unpreferred; (D) Unpreferred-to-Preferred; (E) Spacers in CRM. Black: neutral expectation; Gray: observed site frequency spectrum. (PDF) [file pgen.1002053.s005.pdf]

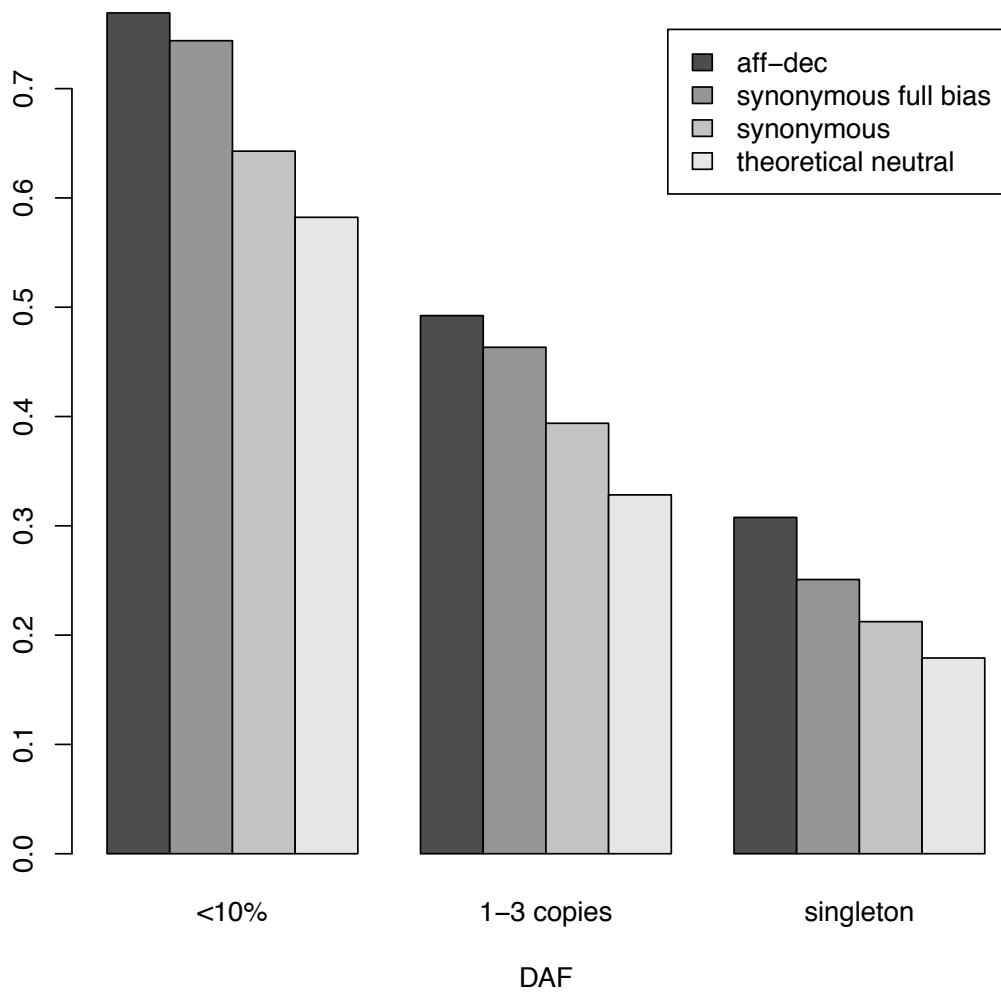

Supplement: Figure S6 — Relative excess of rare variants suggests purifying selection on affinity decreasing mutations in mel. The proportion of low frequency class(es) for affinity-decreasing mutations compared to the theoretical neutral expectation, the observed synonymous sites, or the expected proportion for synonymous sites under ascertainment assuming . DAF: derived allele frequency. (PDF) [file pgen.1002053.s006.pdf]

PWMMel

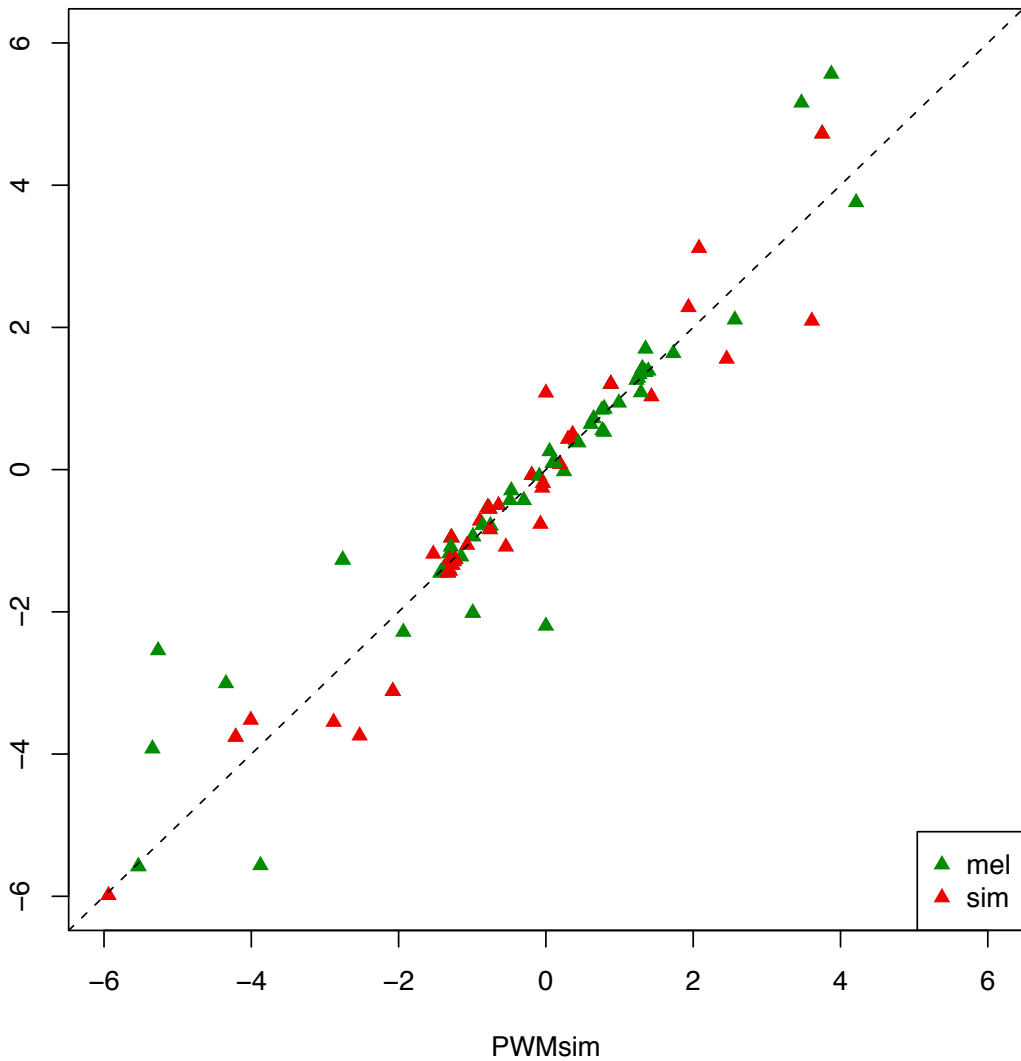

Supplement: Figure S7 — PWM derived from mel footprints (PWMmel) or their aligned sequences in sim (PWMsim) produce consistent results under our classification method. On the scatter plot each point represents a single nucleotide mutation with its x, y values being the estimates of its effect on binding affinity using either the mel PWM or the sim PWM, respectively. Green and red triangles are mutations occurring on mel or sim lineages. From the figure, the PWM have very little biases with respect to scoring mutations from the species where it is derived or the other species. (PDF) [file pgen.1002053.s007.pdf]

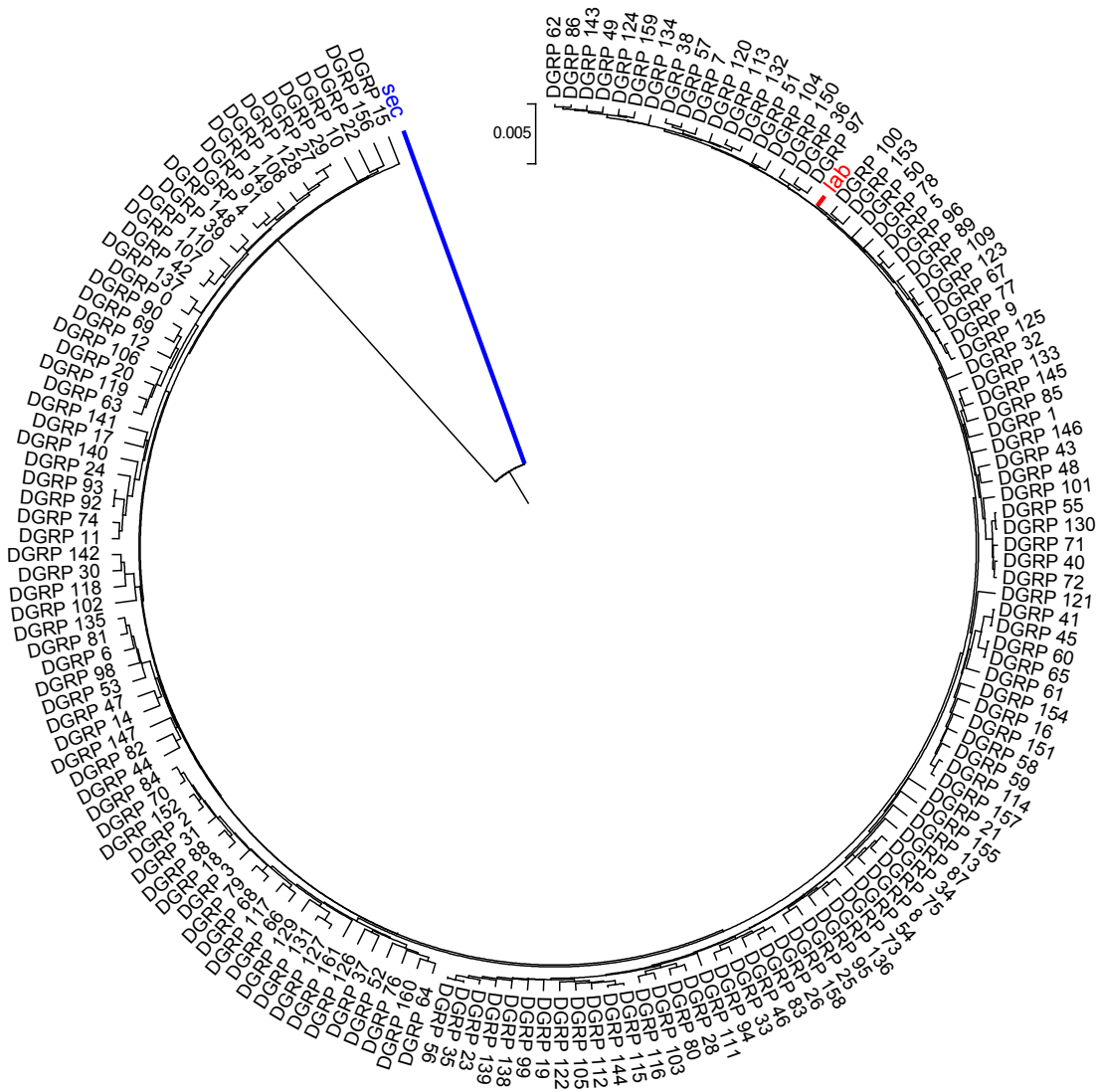

Supplement: Figure S8 — A geneaology tree based on 10 kb CRM sequences for 162 lines from DGRP and the Berkeley reference sequencing strain. The tree is built in MEGA using maximum likelihood method, based on 10 kb sequence alignments. It is rooted with one sequence from a closely related species D. sechellia as an outgroup (bold and blue). The reference sequencing strain (referred to as lab, bold and red) is obviously inter-mingled with the other 162 lines. A similar procedure on 3 different 10 kb sequences sampled from the genome produced similar shaped trees with the lab line embedded among the 162 lines, although the exact orders of branches are not the same, reflecting different geneaologies between regions in the genome. (PDF) [file pgen.1002053.s008.pdf]
