# Supplementary material for: Does Positive Selection Drive Transcription Factor Binding Site Turnover? A Test with Drosophila Cis-Regulatory Modules
Source: PLoS Genet. 2011 Apr 28;7(4):e1002053. doi: 10.1371/journal.pgen.1002053 (PMC3084208; doi:10.1371/journal.pgen.1002053)
Supplement: Table S1 — CRM studied in this study. (PDF) [file pgen.1002053.s009.pdf]

Table S1 CRM studied in this study

name	symbol	Chromosome	start*	end	# footprints
CE1000	E74 Promoter	chr3L	17612224	17612895	8
CE1004	h stripe 6	chr3L	8659948	8660496	25
CE1006	Fbp1 enhancer	chr3L	14091229	14091302	2
CE1007	DNA IIA237 enhancer	chr2R	5822784	5823022	2
CE1008	475bp sev enhancer	chrX	10973456	10973933	6
CE1010	dpp 812bp BE/VM enhance	chr2L	2445768	2446580	30
CE1012	h7 element	chr3L	8658176	8659109	41
CE1013	Stripe 3+7 enhancer	chr2R	5863004	5863516	16
CE1014	1.4kb posterior enhancer	chr3R	4526528	4527944	9
CE1016	2.7kb Dfd EAE	chr3R	2611055	2613713	15
CE1018	NK-1 promoter	chr3R	17382901	17383486	2
CE1019	dpp proximal promoter	chr2L	2454605	2454756	4
CE1020	DNApol-alpha180_promot	chr3R	17497407	17497685	4
CE1021	eve promoter	chr2R	5866417	5866922	18
CE1022	tin B enhancer	chr3R	17205669	17206042	3
CE1025	twist ventral activator	chr2R	18933197	18933859	14
CE1026	ChAT 0.3kb 5' region	chr3R	14530829	14531165	2
CE1027	eve MAS	chr2R	5861380	5861582	6
CE1028	ems enhancer element IV	chr3R	9720483	9720788	4
CE1029	iab-2(1.7) enhancer	chr3R	12636228	12637974	11
CE1030	sal enhancer	chr2L	11455638	11456155	18
CE1031	en promoter	chr2R	7415172	7415811	8
CE1032	tin 5' region	chr3R	17203781	17205010	1
CE1034	Ubx BRE enhancer	chr3R	12526644	12527144	17
CE1035	knirps 5' regulatory region	chr3L	20689622	20690740	39
CE1036	eve mesodermal enhancer	chr2R	5872553	5873441	17
CE1037	SRF-A intervein "c" enhan	chr2R	20229766	20229892	1
CE1038	omb wing enhancer	chrX	4280499	4281987	1
CE1039		chr3L	14070015	14072441	16
CE1041	dpp promoter	chr2L	2452152	2452471	1
CE1042	Antp P1 promoter	chr3R	2830803	2831280	3
CE1047	E2f promoter	chr3R	17458890	17459274	5
CE1048	tud promoter	chr2R	17070613	17071035	1
CE1049	Antp P2 promoter	chr3R	2758361	2760662	64
CE1050	gsb_early_enhancer	chr2R	20943848	20944882	19
CE1051	slbo P2	chr2R	20221605	20221785	1
CE1054	otp regulatory region	chr2R	16786018	16787830	9
CE1055	slp1	chr2L	3823118	3824696	9
CE1056	eve stripe 2	chr2R	5865215	5865887	24
CE1057	alpha1 tubulin	chr3R	2912163	2913017	14
CE1058	Ubx promoter	chr3R	12559789	12560360	20
CE1061	Race 533bp enhancer	chr2L	13904731	13905265	3
CE1062	dorsal vessel enhancer	chr2R	20191677	20191999	2
CE1064	Trl promoter	chr3L	14750248	14751686	27
CE1067	so10 enhancer	chr2R	3318590	3319018	8
CE1068	Ubx PRE	chr3R	12589355	12590918	50

CE1069	tin D	chr3R	17209338	17209690	12
CE1073	B3-15 vm1 enhancer	chr2R	20196541	20196901	6
CE1074	tll CD1	chr3R	26676774	26677274	8
CE1075	ftz-f1 promoter	chr3L	18758302	18758908	3
CE1076	zen promoter	chr3R	2579902	2581525	25
CE1077	rho NEE	chr3L	1461790	1462115	10
CE1078	snail promoter	chr2L	15478269	15480270	12
CE1079	Dll NRE	chr2R	20690304	20691195	3
CE1080	vg boundary	chr2R	8776378	8777133	5
CE1081	vg quadrant enhancer	chr2R	8783677	8784481	8
CE1082	vvl autoreg enhancer	chr3L	6778064	6778580	2
CE1084	ph-p1	chrX	2034243	2034683	6
CE1085	ph-d1	chrX	2019326	2019622	5
CE1086	ph-d2	chrX	2018150	2018483	2
CE1088	scs fragment C	chr3R	7775364	7775488	1
CE1089	hairy stripe 3+4	chr3L	8657462	8658374	15
CE1090	Dpt promoter	chr2R	14752946	14753349	4
CE1094	Deb-A	chr2R	8085631	8086424	2
CE1098	Jra promoter	chr2R	5984349	5984683	1
CE1099	Sgs4	chrX	3143318	3144141	30
CE1101	Act 5C proximal promoter	chrX	5795566	5796038	1
CE1102	sim_2.8_Pe	chr3R	8895635	8898460	9
CE1103	Sgs3_promoter	chr3L	11505175	11505337	3
CE1104	Sgs3_63bp_5'region	chr3L	11504674	11504738	2
CE1105	E74_KpnI_RcoRI_intron	chr3L	17580603	17580926	3
CE1106	Eip74Eg_promoter	chr3L	15650460	15650886	9
CE1107	sc_L3/TSM_enhancer	chrX	289651	289904	2
CE1108	sc_SMC_enhancer	chrX	286995	287352	1
CE1109	ko_intronic_enhancer	chr3L	21080606	21081587	3
CE1110	ras_promoter_cBE76	chrX	10638961	10639282	3
CE1112	hsp23 promoter	chr3L	9373335	9373683	9
CE1115	P1 promoter fragment I	chr3R	2827205	2827388	4
CE1116	P1 promoter fragment II	chr3R	2825808	2826148	4
CE1118	P1 promoter fragment IV	chr3R	2823441	2823507	3
CE1121	ftz zebra element	chr3R	2689374	2690045	19
CE1122	ind cis-regulatory	chr3L	15033494	15033709	3
CE1123	Lz_LMEE	chrX	9181194	9181476	1
CE1126	copia LTR	chrX	4178496	4183743	1
CE1127	ftz_USE	chr3R	2683637	2686199	58
CE1128	Cp15 promoter	chr3L	8721445	8721581	3
CE1129	Cf2 promoter	chr2L	4883103	4883234	1
CE1131	sal 1.1 enhancer	chr2L	11454343	11455436	8
CE1132	kni L2 enhancer	chr3L	20699779	20700471	5
CE1133	ct enhancer	chrX	7424257	7424926	7
CE1134	PCNA promoter	chr2R	16150202	16150749	6
CE1135	bab1_intron1	chr3L	1085706	1086298	4
CE1136	bab2_intron1	chr3L	1175395	1175613	2
CE1138	Adh_promoter	chr2L	14614893	14616302	26
CE1140	scs'	chr3R	7788458	7789554	5

CE1141	otu_promoter	chrX	8383414	8383958	3
CE1144	Sxl_intron	chrX	6985658	6986870	2
CE1145	Orb_promoter	chr3R	19105085	19106451	1
CE1146	ovo_promter	chrX	4957413	4958494	6
CE1147	glass_promoter	chr3R	14200149	14200999	2
CE1148	ninaE_promoter	chr3R	15713875	15714387	5
CE1149	en_promoter	chr2R	7415997	7416555	14
CE1150	en_intron	chr2R	7412745	7414506	15
CE1151	U1_promoter	chr3R	19685239	19685733	1
CE1152	ftz_3'_region	chr3R	2691949	2692335	1
CE1155	Actin5C_promoter	chrX	5794681	5795112	4
CE1156	Hsp27 promoter	chr3L	9376508	9376729	1
CE1157	ACE	chr3L	8719259	8719581	8
CE1159	tll_promoter	chr3R	26677662	26678030	8
CE1160	dpp_disk_BS1.1	chr2L	2469788	2471532	5
CE1161	dpp_VRR_intron	chr2L	2455937	2457661	23
CE1162	hb_promoter	chr3R	4520300	4524677	20
CE1164	Ddc_intron_promoter	chr2L	19119460	19123023	32
CE1169	117	chr3R	7799721	7799941	2
CE1170	410	chr3L	11552404	11552514	1
CE1172	11B11	chrX	2071273	2071411	2
CE1173	11G4	chr3R	13210846	13211094	2
CE1174	11G5	chr2R	17085621	17085745	2
CE1175	11F6	chrX	12524387	12524538	1
CE1176	116	chr3R	13520975	13521188	2
CE1177	417	chr3R	21067232	21067420	3
CE1178	110	chr3L	5571882	5572102	2
CE1179	407	chr2R	6202914	6203028	3
CE1181	pbx_extended	chr3R	12598627	12600114	25
CE1182	DNA pol alpha 73	chr3R	23064563	23065162	3
CE1183	tsh enhancer	chr2L	21852963	21853592	16
CE1184	lab550 enhancer	chr3R	2507112	2507664	3
CE1185	Antp_P1_promoter	chr3R	2824411	2825376	9
CE1186	twi promoter	chr2R	18932628	18933070	3
CE1187	white promoter	chrX	2690577	2691909	9
CE1188	white intron	chrX	2689167	2689286	2
CE1189	Ubx promoter	chr3R	12562550	12563348	4
CE1190	Ubx 3'	chr3R	12481453	12481572	3
CE1191	dpp promoter	chr2L	2450830	2450949	3
CE1192	zeste promoter	chrX	2341986	2342105	2
CE1193	Hsp70 promoter	chr3R	7783054	7784301	13
CE1194	hsp26 promoter	chr3L	9370273	9370988	5
CE1195	hsp83 promoter	chr3L	3193048	3193256	1
CE1196	E(spl) promoter	chr3R	21865315	21866073	2
CE1197	ac promoter	chrX	263167	264014	2
CE1198	l(1)sc promoter	chrX	302783	303755	2
CE1199	yp1 promoter	chrX	9947564	9947891	11
CE1200	Ser wing enhancer	chr3R	22997005	22997817	25
CE1201	ems enhancer	chr3R	9723513	9724763	10

CE1204	ftz_neurogenic_enhancer	chr3R	2687125	2689005	4
CE1205	Sxl_promoter	chrX	6986907	6988310	1

* the coordinates are based on dm3
