# Supplementary material for: Does Positive Selection Drive Transcription Factor Binding Site Turnover? A Test with Drosophila Cis-Regulatory Modules
Source: PLoS Genet. 2011 Apr 28;7(4):e1002053. doi: 10.1371/journal.pgen.1002053 (PMC3084208; doi:10.1371/journal.pgen.1002053)
Supplement: Table S2 — Percentage of gain and loss of TFBS predicted by two set of cutoffs. (PDF) [file pgen.1002053.s010.pdf]

Table S2. Percentage of gain and loss of TFBS predicted by two set of cutoffs

Functional category	Cutoff 80% [b]		Cutoff 0	
	number	%	number	%
Conserved	487	75.6	547	84.8
Weak [a]	124	19.3	70	10.9
Loss				
mel	2	0.3	0	0
sim	16	2.5	16	2.5
Gain				
mel	14	2.2	12	1.9
sim	1	0.2	0	0

[a] weak binding sites: due to the arbitrary cutoff applied, for some footprint sites neither *mel* nor *sim* sequence passed the cutoff and are categorized under "weak sites". [b] The 80% cutoff is chosen by ranking all TFBS for a particular TF from high to low by their PWM scores and then taking the 80% quantile.
