# Supplementary material for: Does Positive Selection Drive Transcription Factor Binding Site Turnover? A Test with Drosophila Cis-Regulatory Modules
Source: PLoS Genet. 2011 Apr 28;7(4):e1002053. doi: 10.1371/journal.pgen.1002053 (PMC3084208; doi:10.1371/journal.pgen.1002053)
Supplement: Table S3 — The pattern of excess for aff-dec mutations in sim is robust to choices of cutoff. (PDF) [file pgen.1002053.s011.pdf]

Table S3. The pattern of excess for aff-dec mutations in *sim* is robust to choices of cutoff

SIM	fix	poly#	ratiio	FET p
Coding				
Non	312	438	0.71	9E-09
Syn				
No Chg	162	446	0.36	
P->U	449	1714	0.26	0.002
U->P	319	695	0.46	0.04
CRM (aff-dec)				
all	38	35	1.09	2E-05
anc.score>0	31	35	0.89	1E-04
anc.score>2	28	33	0.85	3E-03
(all dec)*	33	38	0.87	4E-04

* in the first three rows in aff-dec class, aff-dec mutations are called when PWM score change<-1, the 4th row is the same as the third with respect to the inclusion of footprint sites except it uses all mutations that has PWM score change<0
