# Supplementary material for: Does Positive Selection Drive Transcription Factor Binding Site Turnover? A Test with Drosophila Cis-Regulatory Modules
Source: PLoS Genet. 2011 Apr 28;7(4):e1002053. doi: 10.1371/journal.pgen.1002053 (PMC3084208; doi:10.1371/journal.pgen.1002053)
Supplement: Table S4 — TF protein sequence divergence in different functional regions between mel and sim. (PDF) [file pgen.1002053.s012.pdf]

Table S4. TF protein sequence divergence in different functional regions between *mel* and *sim*

Factor	type	L	Div total	DNA Binding	Other domains
abd-A	Antp Homeobox Family	590	0	0	0
Antp	Antp Homeobox Family	378	0	0	0
Ap	Homeobox	469	0	0	0
Bcd	Homeobox	494	7	0	0
Cad	Homeobox	427	6	0	0
Deaf1	SAND+MYND finger	576	2	0	0
Dl	RHD	999	21	*1	0
En	engrailed homeobox family	552	1	0	0
Eve	even-skipped homeobox family	376	3	0	0
Hb	hunchback C2H2-type zinc-finger protein family	758	5	0	0
Kni	nuclear hormone receptor	429	6	0	0
Kr	Kruple C2H2-type zinc-finger protein family	502	6	0	0
Mad	Caudal Homeodomain Family	427	0	0	0
Ovo	C2H2 zinc finger	1351	30	0	0
Pan	HMG box	751	18	0	
Prd	Homeobox	613	4	0	0
Slbo	bzip	449	7	0	0
Tin	Homeobox	416	7	0	0
Tll	Nuclear Receptor	452	1	0	0
Trl	BTB domain	581	0	0	0
Twi	bHLH	490	7	0	0
Vvl	Homeobox	427	4	0	0
z (zeste)	(unannotated)	575	8	0	0
Zen	Homeobox	353	19	0	0

* Asp(D)->Glu(E) in the RHD domain (DNA binding) between *mel* and *sim*
