# Supplementary material for: Does Positive Selection Drive Transcription Factor Binding Site Turnover? A Test with Drosophila Cis-Regulatory Modules
Source: PLoS Genet. 2011 Apr 28;7(4):e1002053. doi: 10.1371/journal.pgen.1002053 (PMC3084208; doi:10.1371/journal.pgen.1002053)
Supplement: Table S5 — PWM derived from SELEX sequences using MEME. (PDF) [file pgen.1002053.s013.pdf]

Table S5. PWM derived from SELEX sequences using MEME

				best	SELEX Matrix		Footprint Matrix	
**Factor**	**Min**	**Max**	**L**	**round**	Sensitivity	Specificity	Sensitivity	Specificity
Kr	10	10	10	4(2)	0.75	3.97	0.77	7.85
bcd	8	8	8	3(2)	0.89	3.97	0.89	10.02
cad	8	8	8	2	0.69	4	0.46	20.83
hb	9	12	11	3	0.87	3.72	0.98	11.55
prd	8	12	11	3	0.81	3.09	1	10.36
twi	8	12	12	3	1	39.13	0.8	25.25

[a]cutoff for calling a binding sites is chosen to be 0 for the PWM score

[b]number of total predicted hits per footprint site hit (a PWM match within footprint region)
