# Supplementary material for: Does Positive Selection Drive Transcription Factor Binding Site Turnover? A Test with Drosophila Cis-Regulatory Modules
Source: PLoS Genet. 2011 Apr 28;7(4):e1002053. doi: 10.1371/journal.pgen.1002053 (PMC3084208; doi:10.1371/journal.pgen.1002053)
Supplement: Text S1 — Computational prediction of TFBS in CRM. (DOC) [file pgen.1002053.s014.doc]

## Text S1

## Computational prediction of TFBS in CRM

To test the possibility of systematic, directional evolution of TFBS in *mel* and *sim*, we used patser v3e (Gerald Hertz, 2002) to scan *hunchback* (*hb*) regulated CRM (with at least one footprint *hb* TFBS present) in both species. To balance the sensitivity (proportion of footprint TFBS recovered in prediction) and the specificity (additional predicted sites that don’t overlap a footprint), we plotted these two measures over a range of cutoffs and chose one that recovered 91.8% of the footprint sites while predicting nearly twice as many (1.94 times) sites. Each TFBS was then aligned in the two species and classified according to whether it was *mel­*-specific, *sim­*-specific, or shared. The patser scan extended 200bp beyond each border of the CRM (as identified in REDfly) when alignments were available to allow for the possibility of a shift in the CRM boundary in *sim*. The same procedure was repeated for *bicoid (bcd)* and *Krüpple (Kr)*.
