# Supplementary material for: Does Positive Selection Drive Transcription Factor Binding Site Turnover? A Test with Drosophila Cis-Regulatory Modules
Source: PLoS Genet. 2011 Apr 28;7(4):e1002053. doi: 10.1371/journal.pgen.1002053 (PMC3084208; doi:10.1371/journal.pgen.1002053)
Supplement: Text S2 — Neutral expectations for the MK test and the site frequency spectrum under ascertainment. (DOC) [file pgen.1002053.s015.doc]

## Text S2

## Neutral expectations for the MK test and the site frequency spectrum under ascertainment

The ascertainment of footprint TFBS exclusively in *mel* may alter the neutral expectations for MK test and site frequency spectrum in and only in *mel*. This arises as a result of the mutations in *mel* being sampled conditioned on the TFBS being detected in the same species. Since affinity-increasing and affinity-decreasing mutations have the potential to change the detectability of the TFBS as a footprint, the conditioned expectation for the neutral pattern is different from the unascertained case. Where there is a fixed or segregating mutation in *mel* that changes the binding affinity, we assumed that the high-affinity allele is detected with probability 1 while the low affinity one with probability *f*. Applying these assumptions, we calculate the expected number of fixed mutations in the ascertained sample under neutrality, as well as the number of segregating mutations in each frequency classes for the affinity-increasing and affinity-decreasing mutations.

For affinity decreasing mutations, the expected number of segregating mutations at frequency *j* out of *n* in the population sample is

(Ewens 2004, equation 9.17, [1])

$\xi_{j}=\frac{\theta}{j}$, where $\theta=4N\mu$ (1)

In the ascertained sample, however, the number of $\xi_{j}^{*}$ is expected to be

$\xi_{j,dec}^{*}=\xi_{j}\times(1-\frac{j}{n}\times f)$ (2)

where the factor in parenthesis is the probability of sampling the derived allele multiplied by the probability that the TFBS is not detectable, which gives the expected frequency spectrum for affinity-decreasing mutations under ascertainment. For MK table, let *D* be the actual number of mutations fixed in *mel*, of which *D*f* will not be detectable in the ascertained sample. We take the observed values for divergence (*D_0_*) and common polymorphism (*P_0_*, derived allele frequency > 0.15) for the synonymous No-Change class to estimate the expected neutral substitution-to-polymorphism ratio under ascertainment

$R_{c,dec}^{*}(d:p)=\frac{D_{0}\times(1-f)}{\sum_{j>n*0.15}^{n-1} \xi_{j0}\times(1-\frac{j}{n}\times f)}$ (3)

For affinity-increasing mutations, the expected number of mutations segregating at frequency *j/n* in the ascertained sample is

$\xi_{j,inc}^{*}=\xi_{j}\times(1-\frac{n-j}{n}\times f)$ (4)

For MK table, we have

$R_{c,inc}^{*}(d:p)=\frac{D_{0}}{\sum_{j>n*0.15}^{n-1} \xi_{j0}\times(1-\frac{n-j}{n}\times f)}$ (5)

Finally, we attempt to obtain an empirical estimate of *f*. Note that footprint sites for any TF consists of more than a single consensus sequence but spanning a range of affinities. Therefore *f* must be smaller than 1. To estimate *f* for a particular TF, we make the conservative assumption that the lowest PWM score among the footprint sites for that TF is the threshold of the detection limit. We then enumerate all possible single nucleotide mutations (3**L*, *L* is the total number of nucleotides belonging to the TFBS for that TF), and among those predicted to be affinity-decreasing we calculated the proportion $\hat{f}$ which would lead the PWM score of the TFBS to drop below the threshold. Averaging across the 30 TF, we estimated $\hat{f}=0.27\pm0.20$.

[1] W. J. Ewens. *Mathematical Population Genetics*. Springer, 2nd edition, January 2004.
