# Supplementary material for: Does Positive Selection Drive Transcription Factor Binding Site Turnover? A Test with Drosophila Cis-Regulatory Modules
Source: PLoS Genet. 2011 Apr 28;7(4):e1002053. doi: 10.1371/journal.pgen.1002053 (PMC3084208; doi:10.1371/journal.pgen.1002053)
Supplement: Text S3 — Validity of transferring the footprint sites identified in lab strains to the natural populations. (DOC) [file pgen.1002053.s016.doc]

## Text S3

## Validity of transferring the footprint sites identified in lab strains to the natural populations

Are the CRM sequences in the reference genome different from those in the Raleigh, N.C. sample? A concern was raised that laboratory lines might be subject to conditions that may have led to the loss of functional binding sites, either by selection or by genetic drift. If so, the footprint site data on lab liines might not be representative of natural population sequences.

The effect of a non-random choice of lab line with respect to binding affinity can be formulated as follows: in equation (2) and (3) in the Text S2, the *j/n* term assumes that the line used for discovery of the footprint site is chosen randomly from the population. Thus if the lab lines tend to have lower affinity, assume in the extreme condition that it always contain the lower affinity allele, the term would be 1 instead of *j/n*. In such a case, the frequency spectra will be the same as the unascertained case since the effect of the ascertainment is not frequency dependent. According to equation (3) and (5) in Text S2, the *R_c_(d:p)* of affinity decreasing mutations will be the same as in the unascertained case, while the correction factor for affinity-increasing mutations will be larger than the value we derived. We re-calculated the ascertained *R_c_*(*d:p*) under the extreme condition mentioned above, which shows that the observed excess of substitutions for affinity-increasing mutations is not expected under the ascertainment unless *f* is close or greater than 0.60, which is larger than our estimate of 0.27±0.20. Note further that the assumption of the lab lines always carrying the lower affinity allele would mean an over-estimate of *f* based on the footprint sites. Finally, as we will explain below, it is probably not the case that the lab lines were distinct in sequences from the natural populations.

First, all the footprint sequences from the individual studies that identified them invariably match both the Drosophila reference genome sequence, itself derived from a lab line, as well as the concensus sequence derived from the population genetic sample. The latter match directly addresses the concern that the lab sequences are different from the population sequences.

To further investigate whether the lab sequences are diverged from the population sequences, we concatenated the CRM sequences (10kb total) for the Berkeley reference strain and for each of the 162 Raleigh population strains, and used them to construct a tree based on pairwise differences (See Figure S8). The resulting tree shows that the reference strain sequence falls within one of the population sample subclades, indicating that it is not differentiated from the population sample sequences. Replicating this analysis on several sequences sampled from different parts of the genome confirmed this result. Estimates of sequence diversity among the Raleigh population lines and between these lines and the reference strain sequence confirm their similarity. We should also point out that footprint experiments were carried out in many different lab strains instead of a single strain.

Therefore we conclude that the transfer of functional annotation from lab lines to the natural populations is valid in our study.
